# Mortality Risk Between Ages 11 and 22 Years Among Young People With Neurodisability in England: A National Cohort Study Using Linked Health and Education Data

**DOI:** 10.1111/ppe.70178

**Published:** 2026-07-09

**Authors:** Louise Macaulay, Jennifer Saxton, Tamsin Ford, Stuart Logan, Katie Harron, Ruth Gilbert, Ania Zylbersztejn

**Affiliations:** ^1^ Great Ormond Street Institute of Child Health University College London London UK; ^2^ Department of Psychiatry University of Cambridge Cambridge UK; ^3^ NIHR Applied Research Collaboration for the Southwest University of Exeter Medical School Exeter UK; ^4^ NIHR Great Ormond Street Hospital Biomedical Research Centre Great Ormond Street Hospital for Children NHS Foundation Trust London UK

**Keywords:** adolescent, cohort studies, mortality, neurodevelopmental disorders, transition to adult care, young adult

## Abstract

**Background:**

Mortality risk rises from childhood into early adulthood. Young people with neurodisability (neurological conditions causing functional limitations) may be particularly vulnerable during the transition from paediatric to adult services.

**Objectives:**

To estimate all‐cause and cause‐specific mortality risk between the ages of 11–22 years in young people with and without neurodisability in England.

**Methods:**

In this national cohort study, we used linked health and education records from ECHILD to follow pupils from age 11 between 2008–09 and 2014–15 to age 22. Neurodisability was identified from hospital admission records before age 11, likely capturing more severe conditions, and supplemented with education data for intellectual disability and autism. Gender‐specific crude cumulative mortality risk and relative differences were estimated using Kaplan–Meier curves and Cox models, with cause‐specific risk estimated using competing‐risks methods.

**Results:**

Among 3,601,180 young people, 143,864 (4.0%) had neurodisability. Between the ages of 11–22, 5565 (0.15%) died; 24% had neurodisability. By age 22, cumulative mortality risk was 1.6% (95% confidence interval [CI] 1.4, 1.8) among females with neurodisability versus 0.14% (95% CI 0.13, 0.15) among peers without neurodisability (hazard ratio [HR] 13.88, 95% CI 12.55, 15.35), an absolute risk difference of 1.5% (95% CI 1.3, 1.6). Among males with neurodisability, cumulative risk was 1.3% (95% CI 1.2, 1.4) versus 0.28% (95% CI 0.27, 0.29; HR 5.34, 95% CI 4.94, 5.78) among peers, an absolute risk difference of 1.0% (95% CI 0.9, 1.1). Most deaths among those with neurodisability were from medical causes, with cumulative risks of 1.5% (95% CI 1.3, 1.6; HR 25.87, 95% CI 23.03, 29.07) in females and 1.1% (95% CI 0.96, 1.2; HR 14.70, 95% CI 13.29, 16.26) in males by age 22.

**Conclusions:**

Young people with neurodisability had a higher mortality risk than peers between ages 11–22, largely from medical causes, with variation by gender and neurodisability subgroup.

## 1 Background

Neurodisability encompasses long‐term conditions of the brain and nervous system that cause functional limitations in movement, cognition, communication, hearing, vision, emotion, or behaviour. Conditions include intellectual disability, autism, epilepsy, cerebral palsy, and genetic conditions (e.g., Down syndrome). These conditions frequently co‐occur and are associated with complex, long‐term needs, often requiring specialist paediatric care [1]. Around 1 in 28 primary school children in England have a neurodisability recorded in hospital data, likely an underestimate as not all require hospital care [2].

Compared with peers, young people with neurodisability experience poorer outcomes, including higher rates of school absence and exclusion [3, 4, 5, 6], lower educational attainment [4, 5, 6], and greater health service use [5, 6, 7, 8]. They also face an increased risk of death. Among young people aged 0–24 years, mortality risk is estimated to be around 3 to over 29 times higher in those with intellectual disability [9, 10, 11, 12, 13], and 2–7 times higher in those with autism [9, 14, 15], compared with peers without these conditions, with evidence of increased mortality across other neurodisability conditions [9].

The transition into adulthood is a vulnerable period for young people, during which mortality risk rises [16] independence increases, and educational settings change, alongside movement from paediatric to adult services (typically between ages 16 and 18 years in England, although timing varies) [17]. For young people with neurodisability, this transition is often associated with disruptions in care, reduced continuity, and an increased risk of unmet health needs [17, 18]. Existing studies typically focus on single conditions or broad age ranges, limiting understanding of how mortality risk varies across neurodisability conditions and during this transition period.

We used linked health and education data for the whole of England to investigate gender‐specific mortality risk among young people with neurodisability. We characterised neurodisability recorded before secondary school age (< 11 years), and estimated crude cumulative mortality risk, relative differences, and cause‐specific mortality between ages 11 and 22 years compared with peers without neurodisability, spanning the transition from paediatric to adult services.

## 2 Methods

### 2.1 Study Design and Data Sources

In this descriptive national cohort study [19], we used linked education, health and mortality records for young people in England from the Education and Child Health Insights from Linked Data (ECHILD) database. Education records came from the National Pupil Database (NPD), capturing information on pupils in state‐funded schools (approximately 93% of all pupils), including pupil characteristics and Special Educational Needs and Disability (SEND) provision (additional educational support for pupils with health, learning, or behavioural needs) [20, 21]. Health information came from Hospital Episode Statistics (HES) Admitted Patient Care data, which includes only admissions to National Health Service (NHS)‐funded hospitals, with diagnoses coded using the International Classification of Diseases, 10th revision (ICD‐10). Office for National Statistics (ONS) mortality records, routinely linked to HES by NHS England, provided date and cause of death (coded using ICD‐10 from 2001 onwards) [20]. The linked HES‐ONS dataset additionally captures in‐hospital deaths recorded at discharge, which may include recent deaths undergoing coroner's inquest and awaiting registration that do not appear in ONS data [22]. Health and education records were deterministically linked by NHS England using methods described elsewhere, with high linkage rates that increased over time (from 92% in 1990–91 to 99% in 2004–05) [23]. All analyses follow a pre‐specified protocol [24].

### 2.2 Study Population

We developed a cohort of young people enrolled in state‐funded secondary school (mainstream or specialist) in the spring term of year 7 (start of secondary school, usually aged 11 years) between 2008–09 and 2014–15. Young people were excluded if they did not link to a HESID (unique identifier required for linkage to ONS mortality data), had inconsistent dates of birth between HES and NPD (suggesting linkage or data entry errors), had unknown or missing gender, or died before follow‐up (Figure S1). Follow‐up began on 1 February of year 7 (after the spring census date) and continued until the earliest of death or 1 March 2020 (before school records were affected by the first COVID‐19 lockdown in England), a maximum of 11 years (Figure S2).

### 2.3 Neurodisability

We identified children with neurodisability using hospital and educational records prior to secondary school. Neurodisability was primarily ascertained from hospital admissions data, which typically captures children with more severe or complex conditions. Children were classified as having neurodisability if a relevant diagnosis was recorded during any hospital admission before year 7, using a published ICD‐10 code list developed with clinical input. This included conditions with a high likelihood (> 50%) of neurological impairment and functional limitation, such as intellectual disability, autism, cerebral palsy, epilepsy, and Down syndrome (Table S1) [2].

Some conditions, particularly those managed predominantly in community settings, may be under‐recorded in hospital data [2]. To improve ascertainment, we supplemented hospital records with education data for intellectual disability and autism, the only conditions with relevant indicators in these data. Children were additionally classified as having intellectual disability or autism if the condition was recorded as a SEND need type at any point during primary school (years 1–6; Table S2) [21].

### 2.4 Neurodisability Subgroups

We used the ICD‐10 code list and SEND need types described above to identify 28 neurodisability subgroups, including intellectual disability, autism, cerebral palsy, and epilepsy. Subgroups were not mutually exclusive, as children with neurodisability frequently have multiple co‐occurring conditions [1].

Given the under‐recording of intellectual disability in hospital data, explicit ICD‐10 diagnoses alone capture only a subset of affected children [25]. We therefore used an expanded code list comprising: (i) explicit intellectual disability diagnoses; (ii) “high‐risk” conditions (> 75% probability of intellectual disability, e.g., Down syndrome and Rett syndrome); and (iii) “associated” conditions (30%–75% probability, e.g., cerebral palsy and microcephaly; Table S1), developed through literature review and clinical input, and described elsewhere [18]. Hereafter, “intellectual disability” encompasses this broader group.

### 2.5 Cohort Characteristics

We extracted pupil characteristics at baseline (year 7) from the January school census, including gender, ethnic group, first language, school type (mainstream or specialist), and two proxies of socioeconomic deprivation: Income Deprivation Affecting Children Index (IDACI) quintile and free school meals (FSM) eligibility. Educational needs prior to year 7 were characterised by any recorded SEND provision and the highest level of provision, categorised as special educational needs (SEN) support (school‐based support) or Education, Health and Care Plan (EHCP; intensive local government‐funded provision for children with complex needs) [21]. We described chronic health conditions (excluding neurodisability) recorded in hospital admissions prior to year 7 using a published code list [26].

### 2.6 Outcome: Mortality

The primary outcome was all‐cause mortality, indicated by death recorded at hospital discharge or in ONS death registrations. We used a data extract of deaths up to June 2021, but stopped follow‐up on 1 March 2020, allowing up to 15 months for delayed registrations [27]. The secondary outcome was cause‐specific mortality. Deaths were categorised as medical (non‐injury deaths with a recorded cause) or injury‐related using all recorded underlying causes, according to ICD‐10 codes from a published code list [28]. Deaths missing a recorded cause were treated as a separate category and included deaths recorded only in hospital admissions or ONS deaths with no recorded cause.

### 2.7 Statistical Analysis

#### 2.7.1 All‐Cause Mortality

We described baseline characteristics of pupils in year 7 and estimated the prevalence of neurodisability prior to year 7. Using the Kaplan–Meier estimator, we estimated crude cumulative mortality risk between ages 11 and 22 years for young people with and without neurodisability [29]. Relative differences were estimated using hazard ratios from Cox proportional hazards models [29], adjusted for the academic year in which pupils completed year 7 to account for cohort effects. No additional adjustment was undertaken, as the aim was to quantify overall mortality differences between groups [19]. We assessed proportional hazards assumptions using Schoenfeld residuals. Where violations were identified, time‐varying effects were modelled by including interactions between exposure and follow‐up time intervals in Cox models, with hazard ratios from models without interactions interpreted as average effects over follow‐up [30]. All analyses were stratified by gender and conducted using R version 4.4.0.

#### 2.7.2 Cause‐Specific Mortality

We estimated crude cumulative mortality risk from medical, injury‐related, and missing underlying cause of death using the Aalen‐Johansen estimator, accounting for competing risks between causes. Relative risks between young people with and without neurodisability were compared using multi‐state Cox proportional hazards models [29]. We described the distribution of deaths by cause and, for medical causes, by ICD‐10 chapter of the underlying cause.

#### 2.7.3 Neurodisability Subgroup Mortality

We estimated prevalence and crude cumulative mortality risk across the 28 neurodisability subgroups to describe the distribution of conditions within the cohort and variation in mortality. We compared subgroup prevalence captured in ECHILD with published population‐based data to assess representativeness.

#### 2.7.4 Sensitivity Analyses

Sensitivity analyses assessed the impact of neurodisability case definition on all‐cause mortality, comparing: (i) data source for intellectual disability and autism: hospital records, education records, or both; and (ii) intellectual disability definition, reclassifying cases into mutually exclusive hierarchical tiers of diagnostic certainty: explicit diagnoses, “high‐risk” conditions, and “associated” conditions.

#### 2.7.5 Missing Data

Missing data were low for all characteristics (< 5%; Table 1). As analyses were descriptive and models were adjusted only for the academic year, we did not use multiple imputation. Missing values were retained as a separate category.

**TABLE 1:** Characteristics of young people with and without neurodisability in the study cohort.

Characteristic	Total 3,601,180 (100%)^a^	No Neurodisability 3,457,316 (96%)^a^	Any Neurodisability 143,864 (4.0%)^a^
Gender			
Females	1,747,730 (49%)	1,702,771 (49%)	44,959 (31%)
Males	1,853,450 (51%)	1,754,545 (51%)	98,905 (69%)
Ethnic group			
White	2,863,263 (80%)	2,746,146 (79%)	117,117 (81%)
Asian	312,257 (8.7%)	301,952 (8.7%)	10,305 (7.2%)
Black	167,526 (4.7%)	160,897 (4.7%)	6629 (4.6%)
Chinese	11,305 (0.31%)	11,005 (0.32%)	300 (0.21%)
Mixed	150,906 (4.2%)	145,078 (4.2%)	5828 (4.1%)
Any other ethnic group	44,490 (1.2%)	43,252 (1.3%)	1238 (0.86%)
Missing	51,433 (1.4%)	48,986 (1.4%)	2447 (1.7%)
First language			
English	3,128,212 (87%)	3,000,728 (87%)	127,484 (89%)
Other	452,810 (13%)	439,430 (13%)	13,380 (9%)
Missing	20,158 (0.56%)	17,158 (0.50%)	3000 (2.1%)
Recorded as FSM eligible			
No	2,968,540 (82%)^c^	2,864,000 (83%)^c^	104,540 (73%)^c^
Yes	632,630 (18%)^c^	593,310 (17%)^c^	39,320 (27%)^c^
Missing	^b^	^b^	^b^
IDACI quintile			
Q1 (most deprived)	864,450 (24%)	826,101 (24%)	38,349 (27%)
Q2	727,270 (20%)	696,219 (20%)	31,051 (22%)
Q3	676,030 (19%)	649,587 (19%)	26,443 (18%)
Q4	667,397 (19%)	643,713 (19%)	23,684 (16%)
Q5 (least deprived)	648,763 (18%)	627,473 (18%)	21,290 (15%)
Missing	17,270 (0.48%)	14,223 (0.41%)	3047 (2.1%)
Year 7 school			
Mainstream	3,537,481 (98%)	3,434,433 (99%)	103,048 (72%)
Special	63,699 (1.8%)	22,883 (0.66%)	40,816 (28%)
Ever any SEND provision			
No	2,288,591 (64%)	2,268,191 (66%)	20,400 (14%)
Yes	1,312,589 (36%)	1,189,125 (34%)	123,464 (86%)
Highest SEND provision			
SEN support	1,183,684 (33%)	1,131,059 (33%)	52,625 (37%)
EHCP	128,905 (3.6%)	58,066 (1.7%)	70,839 (49%)
Chronic condition (excluding neurodisability)			
No	3,006,601 (83%)	2,933,272 (85%)	73,329 (51%)
Yes	594,579 (17%)	524,044 (15%)	70,535 (49%)

Abbreviations: EHCP, education, health and care plan; FSM, free school meals; IDACI, income deprivation affecting children index; SEN, special educational needs; SEND, special educational needs and disabilities.

^a^ *n* (% of all).

^b^ Counts are suppressed where *n* < 10 to protect confidentiality.

^c^ Counts are rounded to the nearest 10 to protect confidentiality, and percentages are calculated from rounded counts. Totals may not sum exactly due to rounding.

## 3 Results

### 3.1 Cohort Overview

The initial cohort comprised 3,868,256 young people aged 10–12 years enrolled in year 7. After exclusion of those with inconsistent dates of birth between HES and NPD records (approximately 6800, 0.18%), those who did not link to a HESID (approximately 260,210, 6.7%), and those who died before follow‐up or had missing gender (approximately 70, < 0.01%; Figure S1), the final cohort comprised 3,601,180 young people (51% male) with a median follow‐up of 8.1 years (interquartile range 6.1, 10.1). Compared with the final cohort, young people without a HESID were more likely to be from minority ethnic groups, have a first language other than English, and be less likely to have any SEND provision (Table S3).

Of the final cohort, 4.0% had a neurodisability record (69% male). Young people with neurodisability were more likely to be male and socioeconomically deprived, and had substantially higher rates of SEND provision, special school attendance, and co‐occurring chronic conditions than peers (Table 1).

### 3.2 All‐Cause Mortality

**FIGURE 1:**
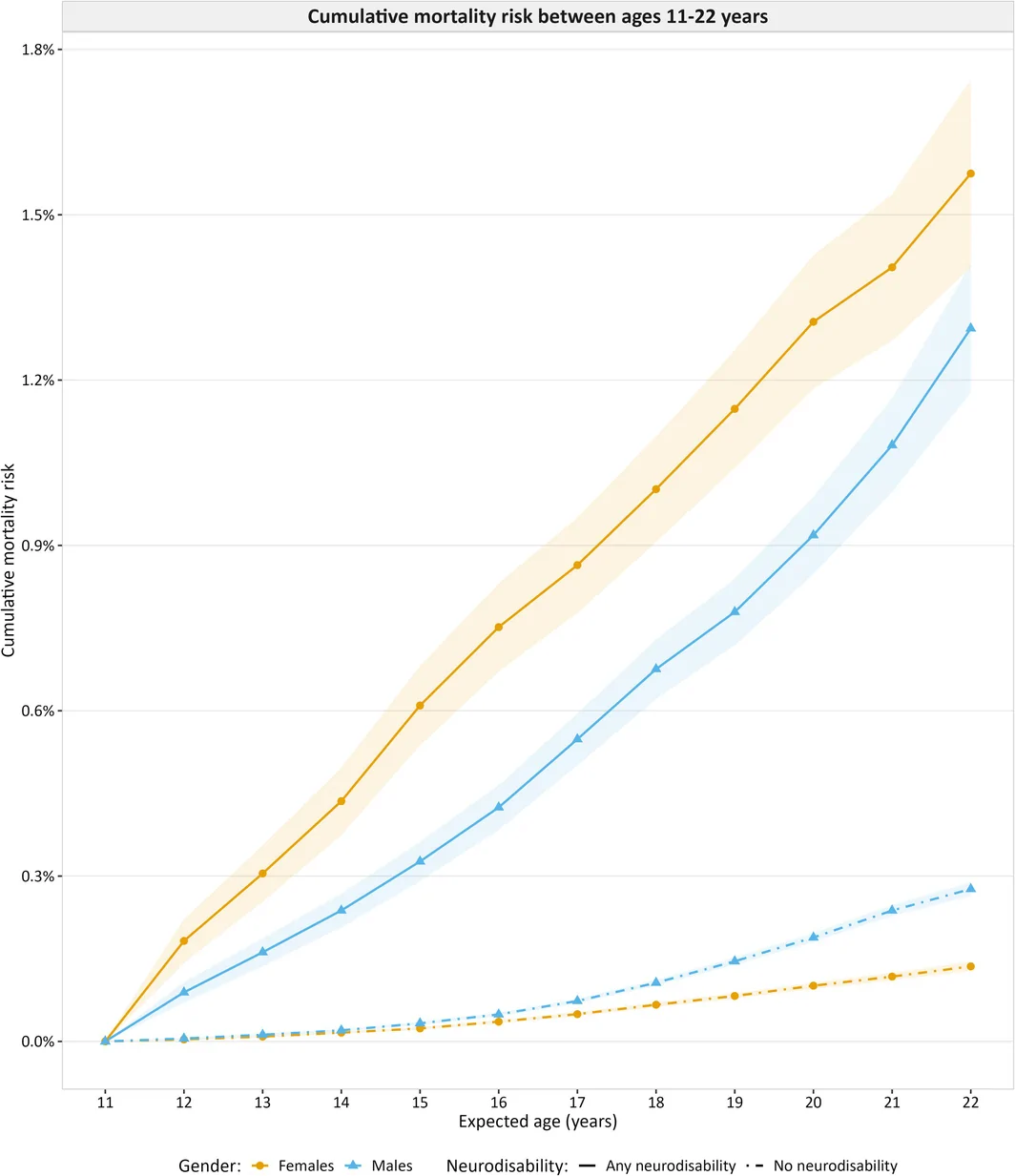
All‐cause cumulative mortality risk between the ages of 11 and 22 years for young people with and without neurodisability. Expected age (years) represents approximate age by school year from the start of year 7 follow‐up (pupils aged 10–12 years at baseline).

Between ages 11 and 22 years, 5565 young people (0.15% of the cohort) had died, of whom nearly one‐quarter had a neurodisability. Among those with neurodisability, around two‐thirds attended special schools, and over four‐fifths had statutory support plans (EHCPs). Cumulative mortality risk in females with neurodisability was 1.6% (95% confidence interval [CI] 1.4, 1.8) compared with 0.14% (95% CI 0.13, 0.15) in females without neurodisability, a 14‐fold higher risk. In males, cumulative risk was 1.3% (95% CI 1.2, 1.4) in those with neurodisability compared with 0.28% (95% CI 0.27, 0.29) in peers, a 5‐fold higher risk (Figure 1 and Table 2).

**TABLE 2:** All‐cause cumulative mortality risk between the ages of 11 and 22 years, absolute risk differences, and hazard ratios for young people with and without neurodisability.

	Females		Males	
	No neurodisability	Any neurodisability	No neurodisability	Any neurodisability
Total deaths	1471	514	2779	801
Cumulative mortality risk (95% CI)	0.14% (0.13, 0.15)	1.6% (1.4, 1.8)	0.28% (0.27, 0.29)	1.3% (1.2, 1.4)
Absolute risk difference (95% CI)	0.00% (Reference)	1.5% (1.3, 1.6)	0.00% (reference)	1.0% (0.9, 1.1)
Hazard Ratio^a^ (95% CI)	1.00 (Reference)	13.88 (12.55, 15.35)	1.00 (Reference)	5.34 (4.94, 5.78)

Abbreviation: CI, confidence interval.

^a^ Adjusted for academic year; hazard ratios represent averages over follow‐up due to time‐varying effects.

The proportional hazards assumption was violated, with hazard ratios highest in early adolescence and generally lower in early adulthood. Hazard ratios were 51.41 (95% CI 36.91, 71.61) for females and 16.81 (95% CI 12.6, 22.5) for males at ages 11–12 years, compared with 6.57 (95% CI 3.66, 11.77) for females at age 20–21 years and 5.08 (95% CI 3.39, 7.63) for males at ages 21–22 years (Figure S3). Therefore, the reported overall hazard ratios represent weighted averages of time‐varying effects over the follow‐up period [30].

### 3.3 Cause‐Specific Mortality

Among young people with neurodisability, most deaths between ages 11 and 22 years were from medical causes (91% in females, 85% in males), compared with approximately half in females and less than one‐third in males without neurodisability. Cumulative mortality risk from medical causes was 1.5% (95% CI 1.3, 1.6) in females with neurodisability compared with 0.07% (95% CI 0.06, 0.07) in peers, corresponding to a nearly 26‐fold higher risk. In males, cumulative risks were 1.1% (95% CI 0.96, 1.2) in those with neurodisability, compared with 0.08% (95% CI 0.07, 0.08) in peers, corresponding to a nearly 15‐fold higher risk (Table 3). Among medical deaths in young people with neurodisability, the most common underlying causes by ICD‐10 chapter were diseases of the nervous system (27% in females, 35% in males), congenital malformations (14% in females, 11% in males), respiratory system diseases (12% in females, 11% in males), and neoplasms (10% in females, 13% in males). By contrast, medical deaths in peers were led by neoplasms (40% in both) and diseases of the circulatory system (14% in females, 19% in males; Table S4).

**TABLE 3:** Cause‐specific cumulative mortality risk between the ages of 11 and 22 years, absolute risk differences, and hazard ratios for young people with and without neurodisability.

Cause of death	Females		Males	
	No neurodisability	Any neurodisability	No neurodisability	Any neurodisability
All	1471	514	2779	801
Medical				
Deaths (%)^a^	714 (49%)	466 (91%)	854 (31%)	681 (85%)
Cumulative mortality risk (95% CI)	0.07% (0.06, 0.07)	1.5% (1.3, 1.6)	0.08% (0.07, 0.08)	1.1% (0.96, 1.2)
Absolute risk difference (95% CI)	0.00% (reference)	1.4% (1.2, 1.6)	0.00% (reference)	1.0% (0.88, 1.1)
Hazard ratio^b^ (95% CI)	1.00 (reference)	25.87 (23.03, 29.07)	1.00 (reference)	14.70 (13.29, 16.26)
Injury				
Deaths (%)^a^	595 (40%)	35 (6.8%)	1555 (56%)	92 (11%)
Cumulative mortality risk (95% CI)	0.05% (0.05, 0.06)	0.10% (0.07, 0.14)	0.15% (0.15, 0.16)	0.16% (0.12, 0.21)
Absolute risk difference (95% CI)	0.00% (reference)	0.05% (0.01, 0.08)	0.00% (reference)	0.01% (−0.04, 0.05)
Hazard ratio^b^ (95% CI)	1.00 (reference)	2.34 (1.67, 3.30)	1.00 (reference)	1.10 (0.89, 1.36)
Missing				
Deaths (%)^a^	162 (11%)	13 (2.5%)	370 (13%)	28 (3.5%)
Cumulative mortality risk (95% CI)	0.02% (0.02, 0.03)	0.04% (0.02, 0.08)	0.05% (0.04, 0.05)	0.08% (0.05, 0.12)
Absolute risk difference (95% CI)	0.00 (reference)	0.02% (0.00, 0.05)	0.00 (reference)	0.03% (−0.01, 0.07)
Hazard ratio^b^ (95% CI)	1.00 (reference)	3.03 (1.72, 5.33)	1.00 (reference)	1.35 (0.92, 1.98)

Abbreviation: CI, confidence interval.

^a^ *n* (% of all deaths).

^b^ Adjusted for academic year; hazard ratios represent averages over follow‐up due to time‐varying effects.

Injury‐related causes accounted for only 6.8% of deaths in females and 11% in males with neurodisability, compared with 40% and 56% in peers, respectively. Cumulative injury‐related mortality risk in females with neurodisability was double that of peers, while males with and without neurodisability had similar risks (Table 3).

Cause of death information was more complete for those with neurodisability (missing 2.5% in females, 3.5% in males) than for peers (missing 11% in females, 13% in males). Cumulative risk for deaths with missing underlying cause was low but higher in those with neurodisability (Table 3).

### 3.4 Neurodisability Subgroup Mortality

**FIGURE 2:**
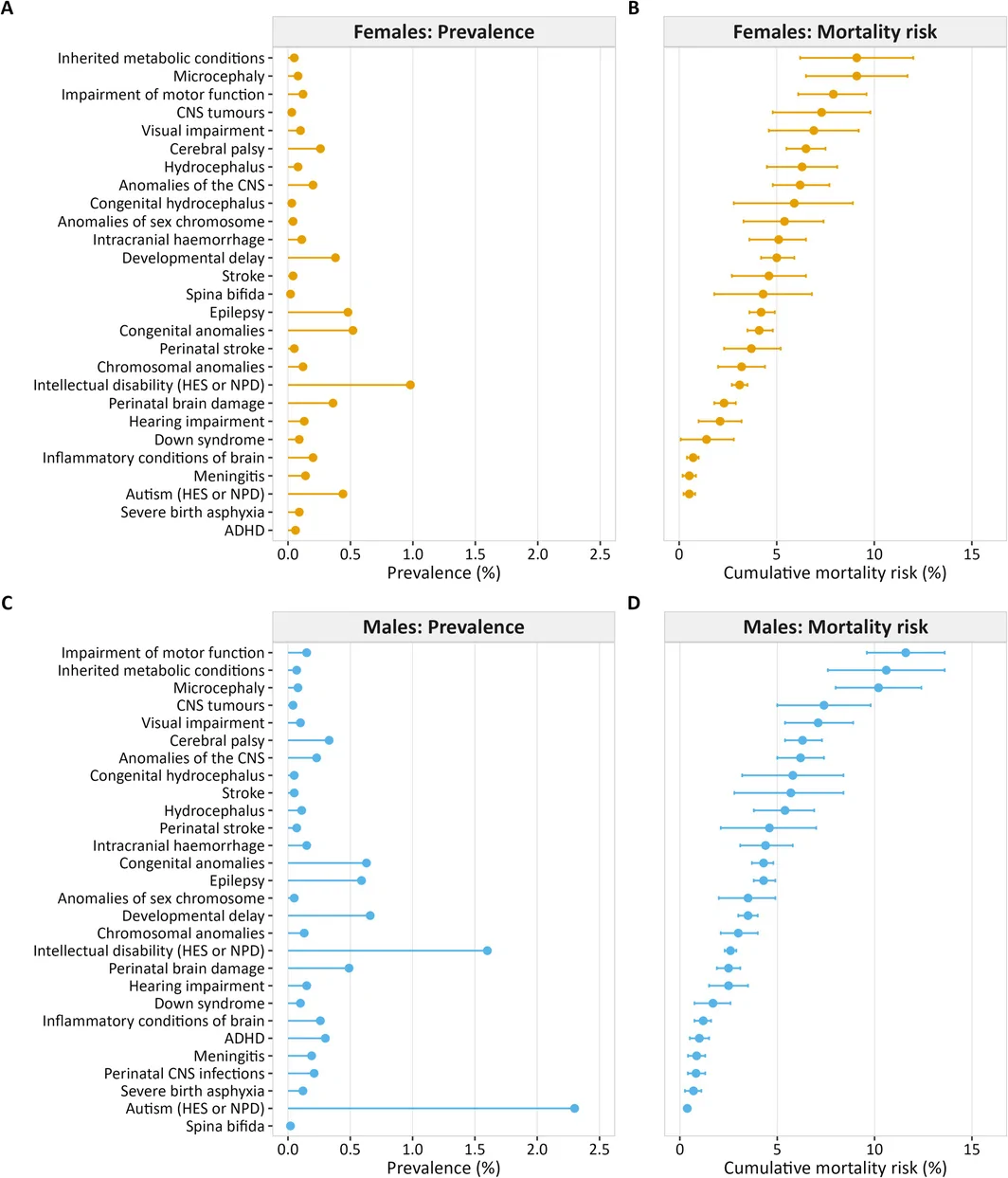
Prevalence and cumulative mortality risk between the ages of 11 and 22 years for neurodisability subgroups. Mortality risk estimates not calculated where deaths < 10. Neurodisability subgroups are non‐mutually exclusive. Intellectual disability refers to cases defined using the primary case definition, including explicit diagnoses, high‐risk conditions, and associated conditions. All conditions identified from HES data unless otherwise stated. ICD‐10 codes and SEND types used to identify conditions are described in Table S1 and Table S2, respectively. ADHD, attention deficit hyperactivity disorder; CNS, central nervous system; HES, hospital episode statistics; ICD‐10, international classification of diseases 10th revision; NPD, National pupil database.

Intellectual disability was the most prevalent subgroup in females (0.98% in females, 1.6% in males), whereas autism was the most prevalent subgroup in males (0.44% in females, 2.3% in males). Other common subgroups included congenital anomalies (including Down syndrome and central nervous system anomalies), epilepsy, and cerebral palsy. For most subgroups, prevalence was higher in males than in females (Figure 2 and Table S5). Prevalence estimates were broadly consistent with published data for conditions typically requiring hospital care, such as cerebral palsy and epilepsy, but were lower than population‐based estimates for conditions more commonly identified in community or educational settings, including intellectual disability and autism (Table S6).

Cumulative mortality risk between ages 11 and 22 years varied substantially across neurodisability subgroups. Among the described prevalent subgroups, cumulative risk was high among young people with cerebral palsy (6.5%, 95% CI 5.5, 7.5 in females; 6.3%, 95% CI 5.4, 7.3 in males), epilepsy (4.2%, 95% CI 3.6, 4.9 in females; 4.3%, 95% CI 3.8, 4.9 in males), congenital anomalies (4.1%, 95% CI 3.5, 4.8 in females; 4.3%, 95% CI 3.7, 4.8 in males), and intellectual disability (3.1%, 95% CI 2.7, 3.5 in females; 2.6%, 95% CI 2.3, 2.9 in males), while risk was low among those with autism (0.52%, 95% CI 0.23, 0.82 in females; 0.38%, 95% CI 0.29, 0.46 in males). Full estimates for all subgroups are presented in Figure 2 and Table S5.

### 3.5 Sensitivity Analyses

Among young people with intellectual disability, cumulative mortality risk was highest in those identified through both hospital and education records (10.1%, 95% CI 8.5, 11.6 in females; 9.3%, 95% CI 8.1, 10.5 in males), followed by hospital records (1.3%, 95% CI 0.88, 1.7 in females; 1.6%, 95% CI 1.2, 2.0 in males), and education records (1.1%, 95% CI 0.81, 1.4 in females; 1.1%, 95% CI 0.83, 1.3 in males). For autism, cumulative risks were highest in males identified from hospital records (1.3%, 95% CI 0.69, 1.9), then both sources (0.51%, 95% CI 0.29, 0.73), and lowest from education records (0.28%, 95% CI 0.19, 0.37). Small death counts limited comparison for females with autism (Table S7).

Among young people with hospital‐recorded intellectual disability, 9.1% of females and 11% of males had an explicit diagnosis, 26% of females and 22% of males had a high‐risk condition, and 65% of females and 67% of males had an associated condition. Cumulative mortality risk was highest in those with an explicit diagnosis (10.9%, 95% CI 7.7, 14.0 in females; 7.3%, 95% CI 5.4, 9.1 in males), followed by associated conditions (4.1%, 95% CI 3.3, 4.8 in females; 4.7%, 95% CI 4.0, 5.3 in males), and lowest in high‐risk conditions (3.7%, 95% CI 2.5, 5.0 in females; 2.9%, 95% CI 2.1, 3.7 in males; Table S8).

## 4 Comment

### 4.1 Principal Findings

In this national cohort of 3.6 million young people in England, 4.0% with neurodisability accounted for nearly one‐quarter of deaths between ages 11 and 22 years. Mortality was substantially higher in young people with neurodisability compared with peers, with larger relative and absolute differences observed among females. Most deaths were due to medical causes, particularly nervous system and respiratory system diseases, congenital malformations, and neoplasms.

### 4.2 Strengths of the Study

ECHILD's scale and coverage enabled 11 years of follow‐up for 3.6 million young people, with sufficient power to examine rare outcomes and stratify by gender and cause of death. Linkage of health and education records provided context on school type and statutory support, highlighting this population's complex needs. Estimation of cumulative mortality risk across neurodisability subgroups extends beyond single‐condition studies, and stratification by cause and gender highlights distinct mortality patterns that can inform targeted prevention.

### 4.3 Limitations of the Data

As a descriptive study, estimates were unadjusted for confounders, reflecting the aim to quantify overall differences but limiting causal interpretation.

Neurodisability was primarily identified from hospital records, which preferentially capture children with more severe or complex conditions. Conditions typically requiring hospital care were therefore better captured, while those managed predominantly in community settings were under‐represented (Table S6). Ascertainment was enhanced using broader code lists and education data for intellectual disability and autism.

We were unable to stratify by condition severity, which likely influences mortality risk and should be considered when interpreting subgroup findings. However, sensitivity analyses showed a lower mortality risk among young people identified solely from education records, consistent with simpler health needs. Co‐occurring conditions may also contribute to observed mortality patterns. Linkage to primary care records would provide more complete coverage of the neurodisability spectrum, but is not currently available within ECHILD.

Cause of death data were incomplete, with 10% of deaths missing a recorded underlying cause, likely reflecting delays in death registration, particularly for deaths requiring an inquest [27]. Cause of death recording was more complete for young people with neurodisability than for peers, likely reflecting higher rates of in‐hospital deaths. We allowed for a 15‐month registration lag to minimise undercounting.

### 4.4 Interpretation

Mortality risk was elevated among both males and females with neurodisability, with larger relative and absolute differences among females, reversing the usual gender pattern in the general population [16]. This partly reflects lower baseline mortality in females without neurodisability, but may also reflect differences in case ascertainment or severity. For example, neurodevelopmental conditions such as autism are often under‐recognised in females and identified later or only in those with more complex presentations [31, 32]. Diagnostic overshadowing may also contribute to delayed recognition of physical health needs [33]. Higher hazard ratios at younger ages for males and females likely reflect lower mortality among peers without neurodisability, although mortality risk remained elevated across all ages.

Mortality varied across neurodisability subgroups. Elevated mortality estimates for young people with conditions such as intellectual disability, cerebral palsy, and epilepsy were broadly consistent with previous population‐based studies [6, 9, 11, 12, 34], while differences across studies, particularly for conditions such as autism [14, 35], likely reflect variation in case ascertainment and population definitions. For intellectual disability, mortality risk increased with complexity of ascertainment source, consistent with greater clinical need. For autism, risk was highest in males identified from hospital records, rather than those identified from both hospital and education records. This may reflect differences in how co‐occurring conditions are captured across health and education records, with some young people identified with autism in hospital data classified under potentially more severe co‐occurring conditions in education data, or educational identification may reflect a less clinically severe group.

Among young people with neurodisability, medical causes dominated, with deaths most commonly from neurological conditions, congenital malformations, respiratory disease, and cancers. Peers died predominantly from injury in males and from a mix of injury and medical causes in females, with medical deaths led by cancers and circulatory disease. The prominence of respiratory disease in the neurodisability population is consistent with evidence that respiratory complications are a leading avoidable cause of death in people with conditions such as intellectual disability and cerebral palsy [36, 37]. These patterns reflect complications of underlying conditions, including seizures, aspiration, and complex co‐occurring conditions [11, 12, 37, 38, 39]. While some deaths reflect progression of severe underlying conditions, others may be amenable to improved recognition of deterioration and better coordination of care [36, 37, 39].

These findings are particularly relevant to the transition from paediatric to adult services, often associated with reduced continuity of care [17]. Young people with neurodisability frequently rely on multiple services, and care fragmentation during transition may increase vulnerability to adverse outcomes. The high level of educational support needs among those who died suggests educational settings may provide important opportunities for coordinated health and education support. Studies including less severe neurodevelopmental conditions are needed to capture the full spectrum of risk, and mechanisms driving gender differences and condition‐specific variation warrant further investigation.

## 5 Conclusions

Young people with neurodisability, identified primarily through hospital‐based diagnoses, accounted for a disproportionate share of deaths between the ages of 11 and 22 years. Mortality differences were driven predominantly by medical causes, were more pronounced among females, and varied across neurodisability subgroups. These findings are consistent with the literature highlighting continuity of care and coordinated support during transition to adult services as important contextual factors for those with complex health needs.

## Author Contributions

The study was designed by L.M. and A.Z.; L.M. analysed the data and wrote the first draft of the manuscript. All authors interpreted the data and contributed to subsequent drafts of the manuscript. All authors have seen and approved the final version.

## Funding

This study was supported by the National Institute for Health and Care Research (NIHR) Programme Development Grants (NIHR206946). The views expressed are those of the author(s) and not necessarily those of the NIHR or the Department of Health and Social Care. ECHILD is supported by Administrative Data Research (ADR) UK, an Economic and Social Research Council (part of UK Research and Innovation) programme (ES/V000977/1, ES/X003663/1, ES/X000427/1). RG was supported by Health Data Research (HDR) UK and by a NIHR senior investigator award. Research at UCL Great Ormond Street Institute of Child Health is supported in part by the NIHR Great Ormond Street Hospital Biomedical Research Centre. The funders had no role in study design, data collection and analysis, decision to publish or preparation of the manuscript.

## Ethics Statement

Ethical approval for the ECHILD project was granted by the National Research Ethics Service (17/LO/1494), NHS Health Research Authority Research Ethics Committee (20/EE/0180 and 21/SW/0159), and is overseen by the UCL Great Ormond Street Institute of Child Health's Joint Research and Development Office (20PE16). Permissions to use linked, de‐identified data from HES and the NPD were granted by NHS England (NIC‐381972) and DfE (DR200604.02), respectively.

## Conflicts of Interest

T.F.'s research group receives funding from Place2Be, a third‐sector organisation that provides mental health interventions and training to UK schools, for research methods consultancy. Other authors declare no conflicts of interest.

## Supporting information


**Supporting Information S1:** ICD‐10 Codes and Special Educational Needs and Disability (SEND) types of need.
**Table S1:** ICD‐10 codes used to classify neurodisability, including subgroup definitions and the expanded intellectual disability classification.
**Table S2:** Special Educational Needs and Disability (SEND) types of need used to identify intellectual disability and autism in school records.

Supporting Information S2:
**Table S3:** Characteristics of young people not linked to Hospital Episode Statistics (HES) records.
**Table S4:** Distribution of underlying causes of death by ICD‐10 chapter among deaths due to medical causes.
**Table S5:** Prevalence before age 11 years and cumulative mortality risk between ages 11 and 22 years by neurodisability subgroup.
**Table S6:** Neurodisability subgroup prevalence before age 11 years in the study cohort compared with published population‐based estimates.
**Table S7:** Cumulative mortality risk between ages 11 and 22 years among young people with intellectual disability or autism by ascertainment source.
**Table S8:** Cumulative mortality risk between ages 11 and 22 years among young people with intellectual disability by case definition.
**Figure S1:** Flow diagram of study cohort derivation. Counts are rounded to the nearest 10 to protect confidentiality and percentages are calculated from rounded counts. Totals may not sum exactly due to rounding. Exact counts for the initial and final cohort are reported in the main text.
**Figure S2:** Overview of the study cohorts, expected age at the start of each academic year, and availability of linked data sources. Cells show the expected age at the start of the academic year and the corresponding school year. Study follow‐up begins at entry to secondary school (Year 7, age 11 years). Colours indicate educational stage, as defined in the legend.
**Figure S3:** Time‐varying hazard ratios for mortality by expected age. Hazard ratios were estimated using Cox proportional hazards models with interaction terms between exposure and time intervals to account for non‐proportional hazards. Expected age (years) represents approximate age by school year from the start of Year 7 follow‐up (pupils aged 10–12 years at baseline) and estimates reflect risk within the preceding year. Estimates for females at age 22 are not reported due to small counts.

## Data Availability

The authors have nothing to report. ECHILD data are not shared publicly in line with data‐sharing agreements with NHS Digital and the Department for Education. ECHILD can be accessed by accredited researchers through application via the ECHILD team (www.echild.ac.uk) and the ONS Research Accreditation Panel.
