# Supplementary material for: Mortality Risk Between Ages 11 and 22 Years Among Young People With Neurodisability in England: A National Cohort Study Using Linked Health and Education Data
Source: Paediatr Perinat Epidemiol. 2026 Jul 9;40(6):756–66. doi: 10.1111/ppe.70178 (PMC13522658; doi:10.1111/ppe.70178)
Supplement: Supplementary file 1 [file PPE-40-756-s001.docx]

Supplementary Material 1: ICD-10 Codes and Special Educational Needs and Disability (SEND) types of need

**Table S1. ICD-10 codes used to classify neurodisability, including subgroup definitions and the expanded intellectual disability classification.**

| **ICD-10 code^†^** | **Description** | **Neurodisability subgroup** ^1^ | **Subtype** | **Intellectual disability classification** ^2^ | **Age criteria**^‡^ |
| --- | --- | --- | --- | --- | --- |
| **Neurodevelopmental conditions** | | | | | |
| F70 | Mild mental retardation | Intellectual disability |  | Explicit diagnosis | 0 |
| F71 | Moderate mental retardation | Intellectual disability |  | Explicit diagnosis | 0 |
| F72 | Severe mental retardation | Intellectual disability |  | Explicit diagnosis | 0 |
| F73 | Profound mental retardation | Intellectual disability |  | Explicit diagnosis | 0 |
| F78 | Other mental retardation | Intellectual disability |  | Explicit diagnosis | 0 |
| F79 | Unspecified mental retardation | Intellectual disability |  | Explicit diagnosis | 0 |
| F80 | Specific developmental disorders of speech and language | Developmental delay |  |  | 0 |
| F800 | Specific speech articulation disorder | Developmental delay |  |  | 0 |
| F801 | Expressive language disorder | Developmental delay |  |  | 0 |
| F802 | Receptive language disorder | Developmental delay |  |  | 0 |
| F808 | Other developmental disorders of speech and language | Developmental delay |  |  | 0 |
| F809 | Developmental disorder of speech and language, unspecified | Developmental delay |  |  | 0 |
| F81 | Specific developmental disorders of scholastic skills | Developmental delay |  |  | 0 |
| F810 | Specific reading disorder | Developmental delay |  |  | 0 |
| F811 | Specific spelling disorder | Developmental delay |  |  | 0 |
| F812 | Specific disorder of arithmetical skills | Developmental delay |  |  | 0 |
| F813 | Mixed disorder of scholastic skills | Developmental delay |  |  | 0 |
| F818 | Other developmental disorders of scholastic skills | Developmental delay |  |  | 0 |
| F819 | Developmental disorder of scholastic skills, unspecified | Developmental delay |  |  | 0 |
| F82 | Specific developmental disorder of motor function | Developmental delay |  |  | 0 |
| F83 | Mixed specific developmental disorders | Developmental delay |  |  | 0 |
| F842 | Rett syndrome | Developmental delay |  | High-risk (>75%) | 0 |
| F88 | Other disorders of psychological development | Developmental delay |  |  | 0 |
| F89 | Unspecified disorder of psychological development | Developmental delay |  |  | 0 |
| F840 | Childhood autism | Autism |  |  | 0 |
| F841 | Atypical autism | Autism |  |  | 0 |
| F843 | Other childhood disintegrative disorder | Autism |  |  | 0 |
| F844 | Overactive disorder associated with mental retardation and stereotyped movements | Autism |  |  | 0 |
| F845 | Asperger syndrome | Autism |  |  | 0 |
| F848 | Other pervasive developmental disorders | Autism |  |  | 0 |
| F849 | Pervasive developmental disorder, unspecified | Autism |  |  | 0 |
| F90 | Hyperkinetic disorders | ADHD |  |  | 0 |
| F900 | Disturbance of activity and attention | ADHD |  |  | 0 |
| F901 | Hyperkinetic conduct disorder | ADHD |  |  | 0 |
| F908 | Other hyperkinetic disorders | ADHD |  |  | 0 |
| F909 | Hyperkinetic disorder, unspecified | ADHD |  |  | 0 |
| F91 | Conduct disorders |  |  |  | 0 |
| F910 | Conduct disorder confined to the family context |  |  |  | 0 |
| F911 | Unsocialized conduct disorder |  |  |  | 0 |
| F912 | Socialized conduct disorder |  |  |  | 0 |
| F913 | Oppositional defiant disorder |  |  |  | 0 |
| F918 | Other conduct disorders |  |  |  | 0 |
| F919 | Conduct disorder, unspecified |  |  |  | 0 |
| F92 | Mixed disorders of conduct and emotions |  |  |  | 0 |
| F920 | Depressive conduct disorder |  |  |  | 0 |
| F928 | Other mixed disorders of conduct and emotions |  |  |  | 0 |
| F929 | Mixed disorder of conduct and emotions, unspecified |  |  |  | 0 |
| F951 | Chronic motor or vocal tic disorder |  |  |  | 0 |
| F952 | Combined vocal and multiple motor tic disorder [de la Tourette] |  |  |  | 0 |
| F958 | Other tic disorders |  |  |  | 0 |
| F959 | Tic disorder, unspecified |  |  |  | 0 |
| F984 | Stereotyped movement disorders |  |  |  | 0 |
| F84 | Pervasive developmental disorders |  |  |  | 0 |
|  | | | | | |
| **Complex neurological conditions** | | | | | |
| G80 | Cerebral palsy | Cerebral palsy |  | Associated (30-75%) | 0 |
| G800 | Spastic quadriplegic cerebral palsy | Cerebral palsy |  | Associated (30-75%) | 0 |
| G801 | Spastic diplegic cerebral palsy | Cerebral palsy |  | Associated (30-75%) | 0 |
| G802 | Spastic hemiplegic cerebral palsy | Cerebral palsy |  | Associated (30-75%) | 0 |
| G803 | Dyskinetic cerebral palsy | Cerebral palsy |  | Associated (30-75%) | 0 |
| G804 | Ataxic cerebral palsy | Cerebral palsy |  | Associated (30-75%) | 0 |
| G808 | Other cerebral palsy | Cerebral palsy |  | Associated (30-75%) | 0 |
| G809 | Cerebral palsy, unspecified | Cerebral palsy |  | Associated (30-75%) | 0 |
| G81 | Hemiplegia | Cerebral palsy |  |  | 0 |
| G810 | Flaccid hemiplegia | Cerebral palsy |  |  | 0 |
| G811 | Spastic hemiplegia | Cerebral palsy |  |  | 0 |
| G819 | Hemiplegia, unspecified | Cerebral palsy |  |  | 0 |
| G82 | Paraplegia and tetraplegia | Cerebral palsy |  |  | 0 |
| G820 | Flaccid paraplegia | Cerebral palsy |  |  | 0 |
| G821 | Spastic paraplegia | Cerebral palsy |  |  | 0 |
| G822 | Paraplegia, unspecified | Cerebral palsy |  |  | 0 |
| G823 | Flaccid tetraplegia | Cerebral palsy |  |  | 0 |
| G824 | Spastic tetraplegia | Cerebral palsy |  |  | 0 |
| G825 | Tetraplegia, unspecified | Cerebral palsy |  |  | 0 |
| G83 | Other paralytic syndromes | Cerebral palsy |  |  | 0 |
| G830 | Diplegia of upper limbs | Cerebral palsy |  |  | 0 |
| G831 | Monoplegia of lower limb | Cerebral palsy |  |  | 0 |
| G832 | Monoplegia of upper limb | Cerebral palsy |  |  | 0 |
| G833 | Monoplegia, unspecified | Cerebral palsy |  |  | 0 |
| F803 | Acquired aphasia with epilepsy [Landau-Kleffner] | Epilepsy |  |  | 0 |
| G40 | Epilepsy | Epilepsy |  |  | 0 |
| G400 | Localization-related (focal)(partial) idiopathic epilepsy and epileptic syndromes with seizures of localized onset | Epilepsy |  |  | 0 |
| G401 | Localization-related (focal)(partial) symptomatic epilepsy and epileptic syndromes with simple partial seizures | Epilepsy |  |  | 0 |
| G402 | Localization-related (focal)(partial) symptomatic epilepsy and epileptic syndromes with complex partial seizures | Epilepsy |  |  | 0 |
| G403 | Generalized idiopathic epilepsy and epileptic syndromes | Epilepsy |  |  | 0 |
| G404 | Other generalized epilepsy and epileptic syndromes | Epilepsy |  |  | 0 |
| G405 | Special epileptic syndromes | Epilepsy |  |  | 0 |
| G406 | Grand mal seizures, unspecified (with or without petit mal) | Epilepsy |  |  | 0 |
| G407 | Petit mal, unspecified, without grand mal seizures | Epilepsy |  |  | 0 |
| G408 | Other epilepsy | Epilepsy |  |  | 0 |
| G409 | Epilepsy, unspecified | Epilepsy |  |  | 0 |
| G41 | Status epilepticus | Epilepsy |  |  | 0 |
| G410 | Grand mal status epilepticus | Epilepsy |  |  | 0 |
| G411 | Petit mal status epilepticus | Epilepsy |  |  | 0 |
| G412 | Complex partial status epilepticus | Epilepsy |  |  | 0 |
| G418 | Other status epilepticus | Epilepsy |  |  | 0 |
| G419 | Status epilepticus, unspecified | Epilepsy |  |  | 0 |
| Y460 | Succinimides | Epilepsy |  |  | 0 |
| Y461 | Oxazolidinediones | Epilepsy |  |  | 0 |
| Y462 | Hydantoin derivatives | Epilepsy |  |  | 0 |
| Y463 | Deoxybarbiturates | Epilepsy |  |  | 0 |
| Y464 | Iminostilbenes | Epilepsy |  |  | 0 |
| Y465 | Valproic acid | Epilepsy |  |  | 0 |
| Y466 | Other and unspecified antiepileptics | Epilepsy |  |  | 0 |
|  | | | | | |
| **Congenital / Chromosomal conditions** | | | | | |
| Q90 | Down syndrome | Chromosomal anomalies | Down syndrome | High-risk (>75%) | 0 |
| Q900 | Trisomy 21, meiotic nondisjunction | Chromosomal anomalies | Down syndrome | High-risk (>75%) | 0 |
| Q901 | Trisomy 21, mosaicism (mitotic nondisjunction) | Chromosomal anomalies | Down syndrome | High-risk (>75%) | 0 |
| Q902 | Trisomy 21, translocation | Chromosomal anomalies | Down syndrome | High-risk (>75%) | 0 |
| Q909 | Down syndrome, unspecified | Chromosomal anomalies | Down syndrome | High-risk (>75%) | 0 |
| Q91 | Edwards' syndrome and Patau syndrome | Chromosomal anomalies |  | High-risk (>75%) | 0 |
| Q910 | Trisomy 18, meiotic nondisjunction | Chromosomal anomalies |  | High-risk (>75%) | 0 |
| Q911 | Trisomy 18, mosaicism (mitotic nondisjunction) | Chromosomal anomalies |  | High-risk (>75%) | 0 |
| Q912 | Trisomy 18, translocation | Chromosomal anomalies |  | High-risk (>75%) | 0 |
| Q913 | Edwards' syndrome, unspecified | Chromosomal anomalies |  | High-risk (>75%) | 0 |
| Q914 | Trisomy 13, meiotic nondisjunction | Chromosomal anomalies |  | High-risk (>75%) | 0 |
| Q915 | Trisomy 13, mosaicism (mitotic nondisjunction) | Chromosomal anomalies |  | High-risk (>75%) | 0 |
| Q916 | Trisomy 13, translocation | Chromosomal anomalies |  | High-risk (>75%) | 0 |
| Q917 | Patau syndrome, unspecified | Chromosomal anomalies |  | High-risk (>75%) | 0 |
| Q92 | Other trisomies and partial trisomies of the autosomes, not elsewhere classified | Chromosomal anomalies |  |  | 0 |
| Q920 | Whole chromosome trisomy, meiotic nondisjunction | Chromosomal anomalies |  | High-risk (>75%) | 0 |
| Q921 | Whole chromosome trisomy, mosaicism (mitotic nondisjunction) | Chromosomal anomalies |  | High-risk (>75%) | 0 |
| Q922 | Major partial trisomy | Chromosomal anomalies |  | High-risk (>75%) | 0 |
| Q923 | Minor partial trisomy | Chromosomal anomalies |  | High-risk (>75%) | 0 |
| Q924 | Duplications seen only at prometaphase | Chromosomal anomalies |  | High-risk (>75%) | 0 |
| Q925 | Duplications with other complex rearrangements | Chromosomal anomalies |  | High-risk (>75%) | 0 |
| Q926 | Extra marker chromosomes | Chromosomal anomalies |  |  | 0 |
| Q927 | Triploidy and polyploidy | Chromosomal anomalies |  | High-risk (>75%) | 0 |
| Q928 | Other specified trisomies and partial trisomies of autosomes | Chromosomal anomalies |  | High-risk (>75%) | 0 |
| Q929 | Trisomy and partial trisomy of autosomes, unspecified | Chromosomal anomalies |  | High-risk (>75%) | 0 |
| Q93 | Monosomies and deletions from the autosomes, not elsewhere classified | Chromosomal anomalies |  | High-risk (>75%) | 0 |
| Q930 | Whole chromosome monosomy, meiotic nondisjunction | Chromosomal anomalies |  | High-risk (>75%) | 0 |
| Q931 | Whole chromosome monosomy, mosaicism (mitotic nondisjunction) | Chromosomal anomalies |  | High-risk (>75%) | 0 |
| Q932 | Chromosome replaced with ring or dicentric | Chromosomal anomalies |  | High-risk (>75%) | 0 |
| Q933 | Deletion of short arm of chromosome 4 | Chromosomal anomalies |  | High-risk (>75%) | 0 |
| Q934 | Deletion of short arm of chromosome 5 | Chromosomal anomalies |  | High-risk (>75%) | 0 |
| Q935 | Other deletions of part of a chromosome | Chromosomal anomalies |  | High-risk (>75%) | 0 |
| Q936 | Deletions seen only at prometaphase | Chromosomal anomalies |  | High-risk (>75%) | 0 |
| Q937 | Deletions with other complex rearrangements | Chromosomal anomalies |  | High-risk (>75%) | 0 |
| Q938 | Other deletions from the autosomes | Chromosomal anomalies |  | High-risk (>75%) | 0 |
| Q939 | Deletion from autosomes, unspecified | Chromosomal anomalies |  | High-risk (>75%) | 0 |
| Q00 | Anencephaly and similar malformations | Anomalies of the CNS |  | High-risk (>75%) | 0 |
| Q000 | Anencephaly | Anomalies of the CNS |  | High-risk (>75%) | 0 |
| Q001 | Craniorachischisis | Anomalies of the CNS |  | High-risk (>75%) | 0 |
| Q002 | Iniencephaly | Anomalies of the CNS |  | High-risk (>75%) | 0 |
| Q01 | Encephalocele | Anomalies of the CNS |  | High-risk (>75%) | 0 |
| Q010 | Frontal encephalocele | Anomalies of the CNS |  | High-risk (>75%) | 0 |
| Q011 | Nasofrontal encephalocele | Anomalies of the CNS |  | High-risk (>75%) | 0 |
| Q012 | Occipital encephalocele | Anomalies of the CNS |  | High-risk (>75%) | 0 |
| Q018 | Encephalocele of other sites | Anomalies of the CNS |  | High-risk (>75%) | 0 |
| Q019 | Encephalocele, unspecified | Anomalies of the CNS |  | High-risk (>75%) | 0 |
| Q02 | Microcephaly | Anomalies of the CNS | Microcephaly | Associated (30-75%) | 0 |
| Q03 | Congenital hydrocephalus | Anomalies of the CNS | Congenital hydrocephalus | Associated (30-75%) | 0 |
| Q030 | Malformations of aqueduct of Sylvius | Anomalies of the CNS | Congenital hydrocephalus | Associated (30-75%) | 0 |
| Q031 | Atresia of foramina of Magendie and Luschka | Anomalies of the CNS | Congenital hydrocephalus | Associated (30-75%) | 0 |
| Q038 | Other congenital hydrocephalus | Anomalies of the CNS | Congenital hydrocephalus | Associated (30-75%) | 0 |
| Q039 | Congenital hydrocephalus, unspecified | Anomalies of the CNS | Congenital hydrocephalus | Associated (30-75%) | 0 |
| Q04 | Other congenital malformations of brain | Anomalies of the CNS |  | Associated (30-75%) | 0 |
| Q040 | Congenital malformations of corpus callosum | Anomalies of the CNS |  | Associated (30-75%) | 0 |
| Q041 | Arhinencephaly | Anomalies of the CNS |  | Associated (30-75%) | 0 |
| Q042 | Holoprosencephaly | Anomalies of the CNS |  | Associated (30-75%) | 0 |
| Q043 | Other reduction deformities of brain | Anomalies of the CNS |  | Associated (30-75%) | 0 |
| Q044 | Septo-optic dysplasia | Anomalies of the CNS |  | Associated (30-75%) | 0 |
| Q045 | Megalencephaly | Anomalies of the CNS |  | Associated (30-75%) | 0 |
| Q046 | Congenital cerebral cysts | Anomalies of the CNS |  | Associated (30-75%) | 0 |
| Q048 | Other specified congenital malformations of brain | Anomalies of the CNS |  | Associated (30-75%) | 0 |
| Q049 | Congenital malformation of brain, unspecified | Anomalies of the CNS |  | Associated (30-75%) | 0 |
| Q05 | Spina bifida | Anomalies of the CNS | Spina bifida |  | 0 |
| Q050 | Cervical spina bifida with hydrocephalus | Anomalies of the CNS | Spina bifida |  | 0 |
| Q051 | Thoracic spina bifida with hydrocephalus | Anomalies of the CNS | Spina bifida |  | 0 |
| Q052 | Lumbar spina bifida with hydrocephalus | Anomalies of the CNS | Spina bifida |  | 0 |
| Q053 | Sacral spina bifida with hydrocephalus | Anomalies of the CNS | Spina bifida |  | 0 |
| Q054 | Unspecified spina bifida with hydrocephalus | Anomalies of the CNS | Spina bifida |  | 0 |
| Q055 | Cervical spina bifida without hydrocephalus | Anomalies of the CNS | Spina bifida |  | 0 |
| Q056 | Thoracic spina bifida without hydrocephalus | Anomalies of the CNS | Spina bifida |  | 0 |
| Q057 | Lumbar spina bifida without hydrocephalus | Anomalies of the CNS | Spina bifida |  | 0 |
| Q058 | Sacral spina bifida without hydrocephalus | Anomalies of the CNS | Spina bifida |  | 0 |
| Q059 | Spina bifida, unspecified | Anomalies of the CNS | Spina bifida |  | 0 |
| Q060 | Amyelia | Anomalies of the CNS |  |  | 0 |
| Q061 | Hypoplasia and dysplasia of spinal cord | Anomalies of the CNS |  |  | 0 |
| Q062 | Diastematomyelia | Anomalies of the CNS |  |  | 0 |
| Q063 | Other congenital cauda equina malformations | Anomalies of the CNS |  |  | 0 |
| Q064 | Hydromyelia | Anomalies of the CNS |  |  | 0 |
| Q068 | Other specified congenital malformations of spinal cord | Anomalies of the CNS |  |  | 0 |
| Q069 | Congenital malformation of spinal cord, unspecified | Anomalies of the CNS |  |  | 0 |
| Q078 | Other specified congenital malformations of nervous system | Anomalies of the CNS |  |  | 0 |
| Q079 | Congenital malformation of nervous system, unspecified | Anomalies of the CNS |  |  | 0 |
| E00 | Congenital iodine-deficiency syndrome |  |  | High-risk (>75%) | 0 |
| E000 | Congenital iodine-deficiency syndrome, neurological type |  |  | High-risk (>75%) | 0 |
| E001 | Congenital iodine-deficiency syndrome, myxoedematous type |  |  | High-risk (>75%) | 0 |
| E002 | Congenital iodine-deficiency syndrome, mixed type |  |  | High-risk (>75%) | 0 |
| E009 | Congenital iodine-deficiency syndrome, unspecified |  |  | High-risk (>75%) | 0 |
| Q860 | Fetal alcohol syndrome (dysmorphic) |  |  | Associated (30-75%) | 0 |
| Q970 | Karyotype 47,XXX | Anomalies of sex chromosome |  |  | 0 |
| Q971 | Female with more than three X chromosomes | Anomalies of sex chromosome |  |  | 0 |
| Q972 | Mosaicism, lines with various numbers of X chromosomes | Anomalies of sex chromosome |  |  | 0 |
| Q978 | Other specified sex chromosome abnormalities, female phenotype | Anomalies of sex chromosome |  |  | 0 |
| Q979 | Sex chromosome abnormality, female phenotype, unspecified | Anomalies of sex chromosome |  |  | 0 |
| Q980 | Klinefelter syndrome karyotype 47,XXY | Anomalies of sex chromosome |  | Associated (30-75%) | 0 |
| Q981 | Klinefelter syndrome, male with more than two X chromosomes | Anomalies of sex chromosome |  | Associated (30-75%) | 0 |
| Q982 | Klinefelter syndrome, male with 46,XX karyotype | Anomalies of sex chromosome |  | Associated (30-75%) | 0 |
| Q983 | Other male with 46,XX karyotype | Anomalies of sex chromosome |  | Associated (30-75%) | 0 |
| Q984 | Klinefelter syndrome, unspecified | Anomalies of sex chromosome |  | Associated (30-75%) | 0 |
| Q985 | Karyotype 47,XYY | Anomalies of sex chromosome |  |  | 0 |
| Q986 | Male with structurally abnormal sex chromosome | Anomalies of sex chromosome |  |  | 0 |
| Q987 | Male with sex chromosome mosaicism | Anomalies of sex chromosome |  |  | 0 |
| Q988 | Other specified sex chromosome abnormalities, male phenotype | Anomalies of sex chromosome |  |  | 0 |
| Q989 | Sex chromosome abnormality, male phenotype, unspecified | Anomalies of sex chromosome |  |  | 0 |
| Q992 | Fragile X chromosome | Anomalies of sex chromosome |  | High-risk (>75%) in males / Associated (30-75%) in females | 0 |
| Q998 | Other specified chromosome abnormalities | Anomalies of sex chromosome |  | High-risk (>75%) | 0 |
| Q999 | Chromosomal abnormality, unspecified | Anomalies of sex chromosome |  | High-risk (>75%) | 0 |
| E702 | Disorders of tyrosine metabolism | Inherited metabolic conditions |  |  | 0 |
| E703 | Albinism | Inherited metabolic conditions |  |  | 0 |
| E708 | Other disorders of aromatic amino-acid metabolism | Inherited metabolic conditions |  |  | 0 |
| E709 | Disorder of aromatic amino-acid metabolism, unspecified | Inherited metabolic conditions |  |  | 0 |
| E71 | Disorders of branched-chain amino-acid metabolism and fatty-acid metabolism | Inherited metabolic conditions |  |  | 0 |
| E710 | Maple-syrup-urine disease | Inherited metabolic conditions |  |  | 0 |
| E711 | Other disorders of branched-chain amino-acid metabolism | Inherited metabolic conditions |  |  | 0 |
| E712 | Disorder of branched-chain amino-acid metabolism, unspecified | Inherited metabolic conditions |  |  | 0 |
| E713 | Disorders of fatty-acid metabolism | Inherited metabolic conditions |  |  | 0 |
| E72 | Other disorders of amino-acid metabolism | Inherited metabolic conditions |  |  | 0 |
| E720 | Disorders of amino-acid transport | Inherited metabolic conditions |  |  | 0 |
| E721 | Disorders of sulfur-bearing amino-acid metabolism | Inherited metabolic conditions |  |  | 0 |
| E722 | Disorders of urea cycle metabolism | Inherited metabolic conditions |  |  | 0 |
| E723 | Disorders of lysine and hydroxylysine metabolism | Inherited metabolic conditions |  |  | 0 |
| E724 | Disorders of ornithine metabolism | Inherited metabolic conditions |  |  | 0 |
| E725 | Disorders of glycine metabolism | Inherited metabolic conditions |  |  | 0 |
| E728 | Other specified disorders of amino-acid metabolism | Inherited metabolic conditions |  |  | 0 |
| E729 | Disorder of amino-acid metabolism, unspecified | Inherited metabolic conditions |  |  | 0 |
| E74 | Other disorders of carbohydrate metabolism | Inherited metabolic conditions |  |  | 0 |
| E740 | Glycogen storage disease | Inherited metabolic conditions |  |  | 0 |
| E741 | Disorders of fructose metabolism | Inherited metabolic conditions |  |  | 0 |
| E742 | Disorders of galactose metabolism | Inherited metabolic conditions |  |  | 0 |
| E743 | Other disorders of intestinal carbohydrate absorption | Inherited metabolic conditions |  |  | 0 |
| E744 | Disorders of pyruvate metabolism and gluconeogenesis | Inherited metabolic conditions |  |  | 0 |
| E748 | Other specified disorders of carbohydrate metabolism | Inherited metabolic conditions |  |  | 0 |
| E749 | Disorder of carbohydrate metabolism, unspecified | Inherited metabolic conditions |  |  | 0 |
| E75 | Disorders of sphingolipid metabolism and other lipid storage disorders | Inherited metabolic conditions |  | High-risk (>75%) | 0 |
| E750 | GM_2_ gangliosidosis | Inherited metabolic conditions |  | High-risk (>75%) | 0 |
| E751 | Other gangliosidosis | Inherited metabolic conditions |  | High-risk (>75%) | 0 |
| E752 | Other sphingolipidosis | Inherited metabolic conditions |  | High-risk (>75%) | 0 |
| E753 | Sphingolipidosis, unspecified | Inherited metabolic conditions |  | High-risk (>75%) | 0 |
| E754 | Neuronal ceroid lipofuscinosis | Inherited metabolic conditions |  | High-risk (>75%) | 0 |
| E755 | Other lipid storage disorders | Inherited metabolic conditions |  | High-risk (>75%) | 0 |
| E756 | Lipid storage disorder, unspecified | Inherited metabolic conditions |  | High-risk (>75%) | 0 |
| E76 | Disorders of glycosaminoglycan metabolism | Inherited metabolic conditions |  | Associated (30-75%) | 0 |
| E760 | Mucopolysaccharidosis, type I | Inherited metabolic conditions |  | Associated (30-75%) | 0 |
| E761 | Mucopolysaccharidosis, type II | Inherited metabolic conditions |  | Associated (30-75%) | 0 |
| E762 | Other mucopolysaccharidoses | Inherited metabolic conditions |  | Associated (30-75%) | 0 |
| E763 | Mucopolysaccharidosis, unspecified | Inherited metabolic conditions |  | Associated (30-75%) | 0 |
| E768 | Other disorders of glucosaminoglycan metabolism | Inherited metabolic conditions |  | Associated (30-75%) | 0 |
| E769 | Disorder of glucosaminoglycan metabolism, unspecified | Inherited metabolic conditions |  | Associated (30-75%) | 0 |
| E77 | Disorders of glycoprotein metabolism | Inherited metabolic conditions |  | Associated (30-75%) | 0 |
| E770 | Defects in post-translational modification of lysosomal enzymes | Inherited metabolic conditions |  | Associated (30-75%) | 0 |
| E771 | Defects in glycoprotein degradation | Inherited metabolic conditions |  | Associated (30-75%) | 0 |
| E778 | Other disorders of glycoprotein metabolism | Inherited metabolic conditions |  | Associated (30-75%) | 0 |
| E779 | Disorder of glycoprotein metabolism, unspecified | Inherited metabolic conditions |  | Associated (30-75%) | 0 |
| E791 | Lesch-Nyhan syndrome | Inherited metabolic conditions |  | High-risk (>75%) | 0 |
| E798 | Other disorders of purine and pyrimidine metabolism | Inherited metabolic conditions |  |  | 0 |
| E799 | Disorder of purine and pyrimidine metabolism, unspecified | Inherited metabolic conditions |  |  | 0 |
| E830 | Disorders of copper metabolism | Inherited metabolic conditions |  | High-risk (>75%) | 0 |
| E851 | Neuropathic heredofamilial amyloidosis | Inherited metabolic conditions |  |  | 0 |
| E888 | Other specified metabolic disorders | Inherited metabolic conditions |  | Associated (30-75%) | 0 |
| E889 | Metabolic disorder, unspecified | Inherited metabolic conditions |  | Associated (30-75%) | 0 |
| D821 | Di George syndrome |  |  |  | 0 |
| Q750 | Craniosynostosis |  |  |  | 0 |
| Q85 | Phakomatoses, not elsewhere classified |  |  |  | 0 |
| Q850 | Neurofibromatosis (nonmalignant) |  |  |  | 0 |
| Q851 | Tuberous sclerosis |  |  | Associated (30-75%) | 0 |
| Q858 | Other phakomatoses, not elsewhere classified |  |  | Associated (30-75%) | 0 |
| Q859 | Phakomatosis, unspecified |  |  | Associated (30-75%) | 0 |
| Q861 | Fetal hydantoin syndrome |  |  | Associated (30-75%) | 0 |
| Q862 | Dysmorphism due to warfarin |  |  | Associated (30-75%) | 0 |
| Q868 | Other congenital malformation syndromes due to known exogenous causes |  |  | Associated (30-75%) | 0 |
| Q870 | Congenital malformation syndromes predominantly affecting facial appearance |  |  | Associated (30-75%) | 0 |
| Q871 | Congenital malformation syndromes predominantly associated with short stature |  |  | Associated (30-75%) | 0 |
| Q872 | Congenital malformation syndromes predominantly involving limbs |  |  | Associated (30-75%) | 0 |
| Q873 | Congenital malformation syndromes involving early overgrowth |  |  | Associated (30-75%) | 0 |
| Q875 | Other congenital malformation syndromes with other skeletal changes |  |  | Associated (30-75%) | 0 |
| Q878 | Other specified congenital malformation syndromes, not elsewhere classified |  |  | Associated (30-75%) | 0 |
| Q899 | Congenital malformation, unspecified |  |  |  | 0 |
|  | | | | | |
| **High-risk conditions affecting the brain** | | | | | |
| G91 | Hydrocephalus | Hydrocephalus |  |  | 0 |
| G910 | Communicating hydrocephalus | Hydrocephalus |  |  | 0 |
| G911 | Obstructive hydrocephalus | Hydrocephalus |  |  | 0 |
| G912 | Normal-pressure hydrocephalus | Hydrocephalus |  |  | 0 |
| G913 | Post-traumatic hydrocephalus, unspecified | Hydrocephalus |  |  | 0 |
| G918 | Other hydrocephalus | Hydrocephalus |  |  | 0 |
| G919 | Hydrocephalus, unspecified | Hydrocephalus |  |  | 0 |
| G940 | Hydrocephalus in infectious and parasitic diseases classified elsewhere | Hydrocephalus |  |  | 0 |
| G941 | Hydrocephalus in neoplastic disease | Hydrocephalus |  |  | 0 |
| G942 | Hydrocephalus in other diseases classified elsewhere | Hydrocephalus |  |  | 0 |
| G972 | Intracranial hypotension following ventricular shunting | Hydrocephalus |  |  | 0 |
| T850 | Mechanical complication of ventricular intracranial (communicating) shunt | Hydrocephalus |  |  | 0 |
| Z982 | Presence of cerebrospinal fluid drainage device | Hydrocephalus |  |  | 0 |
| G46 | Vascular syndromes of brain in cerebrovascular diseases | Stroke |  |  | 0 |
| G460 | Middle cerebral artery syndrome | Stroke |  |  | 0 |
| G461 | Anterior cerebral artery syndrome | Stroke |  |  | 0 |
| G462 | Posterior cerebral artery syndrome | Stroke |  |  | 0 |
| G463 | Brain stem stroke syndrome | Stroke |  |  | 0 |
| G464 | Cerebellar stroke syndrome | Stroke |  |  | 0 |
| G465 | Pure motor lacunar syndrome | Stroke |  |  | 0 |
| G466 | Pure sensory lacunar syndrome | Stroke |  |  | 0 |
| G467 | Other lacunar syndromes | Stroke |  |  | 0 |
| G468 | Other vascular syndromes of brain in cerebrovascular diseases | Stroke |  |  | 0 |
| I60 | Subarachnoid haemorrhage | Stroke |  |  | 0 |
| I600 | Subarachnoid haemorrhage from carotid siphon and bifurcation | Stroke |  |  | 0 |
| I601 | Subarachnoid haemorrhage from middle cerebral artery | Stroke |  |  | 0 |
| I602 | Subarachnoid haemorrhage from anterior communicating artery | Stroke |  |  | 0 |
| I603 | Subarachnoid haemorrhage from posterior communicating artery | Stroke |  |  | 0 |
| I604 | Subarachnoid haemorrhage from basilar artery | Stroke |  |  | 0 |
| I605 | Subarachnoid haemorrhage from vertebral artery | Stroke |  |  | 0 |
| I606 | Subarachnoid haemorrhage from other intracranial arteries | Stroke |  |  | 0 |
| I607 | Subarachnoid haemorrhage from intracranial artery, unspecified | Stroke |  |  | 0 |
| I608 | Other subarachnoid haemorrhage | Stroke |  |  | 0 |
| I609 | Subarachnoid haemorrhage, unspecified | Stroke |  |  | 0 |
| I61 | Intracerebral haemorrhage | Stroke |  |  | 0 |
| I610 | Intracerebral haemorrhage in hemisphere, subcortical | Stroke |  |  | 0 |
| I611 | Intracerebral haemorrhage in hemisphere, cortical | Stroke |  |  | 0 |
| I612 | Intracerebral haemorrhage in hemisphere, unspecified | Stroke |  |  | 0 |
| I613 | Intracerebral haemorrhage in brain stem | Stroke |  |  | 0 |
| I614 | Intracerebral haemorrhage in cerebellum | Stroke |  |  | 0 |
| I615 | Intracerebral haemorrhage, intraventricular | Stroke |  |  | 0 |
| I616 | Intracerebral haemorrhage, multiple localized | Stroke |  |  | 0 |
| I618 | Other intracerebral haemorrhage | Stroke |  |  | 0 |
| I619 | Intracerebral haemorrhage, unspecified | Stroke |  |  | 0 |
| I63 | Cerebral infarction | Stroke |  |  | 0 |
| I630 | Cerebral infarction due to thrombosis of precerebral arteries | Stroke |  |  | 0 |
| I631 | Cerebral infarction due to embolism of precerebral arteries | Stroke |  |  | 0 |
| I632 | Cerebral infarction due to unspecified occlusion or stenosis of precerebral arteries | Stroke |  |  | 0 |
| I633 | Cerebral infarction due to thrombosis of cerebral arteries | Stroke |  |  | 0 |
| I634 | Cerebral infarction due to embolism of cerebral arteries | Stroke |  |  | 0 |
| I635 | Cerebral infarction due to unspecified occlusion or stenosis of cerebral arteries | Stroke |  |  | 0 |
| I636 | Cerebral infarction due to cerebral venous thrombosis, nonpyogenic | Stroke |  |  | 0 |
| I638 | Other cerebral infarction | Stroke |  |  | 0 |
| I639 | Cerebral infarction, unspecified | Stroke |  |  | 0 |
| I64 | Stroke, not specified as haemorrhage or infarction | Stroke |  |  | 0 |
| I67 | Other cerebrovascular diseases | Stroke |  |  | 0 |
| I670 | Dissection of cerebral arteries, nonruptured | Stroke |  |  | 0 |
| I671 | Cerebral aneurysm, nonruptured | Stroke |  |  | 0 |
| I672 | Cerebral atherosclerosis | Stroke |  |  | 0 |
| I673 | Progressive vascular leukoencephalopathy | Stroke |  |  | 0 |
| I674 | Hypertensive encephalopathy | Stroke |  |  | 0 |
| I675 | Moyamoya disease | Stroke |  |  | 0 |
| I676 | Nonpyogenic thrombosis of intracranial venous system | Stroke |  |  | 0 |
| I677 | Cerebral arteritis, not elsewhere classified | Stroke |  |  | 0 |
| I678 | Other specified cerebrovascular diseases | Stroke |  |  | 0 |
| I679 | Cerebrovascular disease, unspecified | Stroke |  |  | 0 |
| I680 | Cerebral amyloid angiopathy | Stroke |  |  | 0 |
| I69 | Sequelae of cerebrovascular disease | Stroke |  |  | 0 |
| I690 | Sequelae of subarachnoid haemorrhage | Stroke |  |  | 0 |
| I691 | Sequelae of intracerebral haemorrhage | Stroke |  |  | 0 |
| I692 | Sequelae of other nontraumatic intracranial haemorrhage | Stroke |  |  | 0 |
| I693 | Sequelae of cerebral infarction | Stroke |  |  | 0 |
| I694 | Sequelae of stroke, not specified as haemorrhage or infarction | Stroke |  |  | 0 |
| I698 | Sequelae of other and unspecified cerebrovascular diseases | Stroke |  |  | 0 |
| I780 | Hereditary haemorrhagic telangiectasia | Stroke |  |  | 0 |
| C70 | Malignant neoplasm of meninges | CNS tumours |  |  | 0 |
| C700 | Malignant neoplasm: Cerebral meninges | CNS tumours |  |  | 0 |
| C701 | Malignant neoplasm: Spinal meninges | CNS tumours |  |  | 0 |
| C709 | Malignant neoplasm: Meninges, unspecified | CNS tumours |  |  | 0 |
| C71 | Malignant neoplasm of brain | CNS tumours |  |  | 0 |
| C710 | Malignant neoplasm: Cerebrum, except lobes and ventricles | CNS tumours |  |  | 0 |
| C711 | Malignant neoplasm: Frontal lobe | CNS tumours |  |  | 0 |
| C712 | Malignant neoplasm: Temporal lobe | CNS tumours |  |  | 0 |
| C713 | Malignant neoplasm: Parietal lobe | CNS tumours |  |  | 0 |
| C714 | Malignant neoplasm: Occipital lobe | CNS tumours |  |  | 0 |
| C715 | Malignant neoplasm: Cerebral ventricle | CNS tumours |  |  | 0 |
| C716 | Malignant neoplasm: Cerebellum | CNS tumours |  |  | 0 |
| C717 | Malignant neoplasm: Brain stem | CNS tumours |  |  | 0 |
| C718 | Malignant neoplasm: Overlapping lesion of brain | CNS tumours |  |  | 0 |
| C719 | Malignant neoplasm: Brain, unspecified | CNS tumours |  |  | 0 |
| C72 | Malignant neoplasm of spinal cord, cranial nerves and other parts of central nervous system | CNS tumours |  |  | 0 |
| C720 | Malignant neoplasm: Spinal cord | CNS tumours |  |  | 0 |
| C721 | Malignant neoplasm: Cauda equina | CNS tumours |  |  | 0 |
| C722 | Malignant neoplasm: Olfactory nerve | CNS tumours |  |  | 0 |
| C723 | Malignant neoplasm: Optic nerve | CNS tumours |  |  | 0 |
| C724 | Malignant neoplasm: Acoustic nerve | CNS tumours |  |  | 0 |
| C725 | Malignant neoplasm: Other and unspecified cranial nerves | CNS tumours |  |  | 0 |
| C728 | Malignant neoplasm: Overlapping lesion of brain and other parts of central nervous system | CNS tumours |  |  | 0 |
| C729 | Malignant neoplasm: Central nervous system, unspecified | CNS tumours |  |  | 0 |
| D32 | Benign neoplasm of meninges | CNS tumours |  |  | 0 |
| D320 | Benign neoplasm: Cerebral meninges | CNS tumours |  |  | 0 |
| D321 | Benign neoplasm: Spinal meninges | CNS tumours |  |  | 0 |
| D329 | Benign neoplasm: Meninges, unspecified | CNS tumours |  |  | 0 |
| D33 | Benign neoplasm of brain and other parts of central nervous system | CNS tumours |  |  | 0 |
| D330 | Benign neoplasm: Brain, supratentorial | CNS tumours |  |  | 0 |
| D331 | Benign neoplasm: Brain, infratentorial | CNS tumours |  |  | 0 |
| D332 | Benign neoplasm: Brain, unspecified | CNS tumours |  |  | 0 |
| D333 | Benign neoplasm: Cranial nerves | CNS tumours |  |  | 0 |
| D334 | Benign neoplasm: Spinal cord | CNS tumours |  |  | 0 |
| D337 | Benign neoplasm: Other specified parts of central nervous system | CNS tumours |  |  | 0 |
| D339 | Benign neoplasm: Central nervous system, unspecified | CNS tumours |  |  | 0 |
| D43 | Neoplasm of uncertain or unknown behaviour of brain and central nervous system | CNS tumours |  |  | 0 |
| D430 | Neoplasm of uncertain or unknown behaviour: Brain, supratentorial | CNS tumours |  |  | 0 |
| D431 | Neoplasm of uncertain or unknown behaviour: Brain, infratentorial | CNS tumours |  |  | 0 |
| D432 | Neoplasm of uncertain or unknown behaviour: Brain, unspecified | CNS tumours |  |  | 0 |
| D433 | Neoplasm of uncertain or unknown behaviour: Cranial nerves | CNS tumours |  |  | 0 |
| D434 | Neoplasm of uncertain or unknown behaviour: Spinal cord | CNS tumours |  |  | 0 |
| D437 | Neoplasm of uncertain or unknown behaviour: Other parts of central nervous system | CNS tumours |  |  | 0 |
| D439 | Neoplasm of uncertain or unknown behaviour: Central nervous system, unspecified | CNS tumours |  |  | 0 |
| A066 | Amoebic brain abscess | Inflammatory conditions of the brain |  |  | 0 |
| A17 | Tuberculosis of nervous system | Inflammatory conditions of the brain |  |  | 0 |
| A170 | Tuberculous meningitis | Inflammatory conditions of the brain | Meningitis |  | 0 |
| A171 | Meningeal tuberculoma | Inflammatory conditions of the brain | Meningitis |  | 0 |
| A178 | Other tuberculosis of nervous system | Inflammatory conditions of the brain |  |  | 0 |
| A179 | Tuberculosis of nervous system, unspecified | Inflammatory conditions of the brain |  |  | 0 |
| A203 | Plague meningitis | Inflammatory conditions of the brain | Meningitis |  | 0 |
| A321 | Listerial meningitis and meningoencephalitis | Inflammatory conditions of the brain | Meningitis |  | 0 |
| A390 | Meningococcal meningitis | Inflammatory conditions of the brain | Meningitis |  | 0 |
| A521 | Symptomatic neurosyphilis | Inflammatory conditions of the brain |  |  | 0 |
| A522 | Asymptomatic neurosyphilis | Inflammatory conditions of the brain |  |  | 0 |
| A523 | Neurosyphilis, unspecified | Inflammatory conditions of the brain |  |  | 0 |
| A800 | Acute paralytic poliomyelitis, vaccine-associated | Inflammatory conditions of the brain |  |  | 0 |
| A801 | Acute paralytic poliomyelitis, wild virus, imported | Inflammatory conditions of the brain |  |  | 0 |
| A802 | Acute paralytic poliomyelitis, wild virus, indigenous | Inflammatory conditions of the brain |  |  | 0 |
| A803 | Acute paralytic poliomyelitis, other and unspecified | Inflammatory conditions of the brain |  |  | 0 |
| A809 | Acute poliomyelitis, unspecified | Inflammatory conditions of the brain |  |  | 0 |
| A82 | Rabies | Inflammatory conditions of the brain |  |  | 0 |
| A820 | Sylvatic rabies | Inflammatory conditions of the brain |  |  | 0 |
| A821 | Urban rabies | Inflammatory conditions of the brain |  |  | 0 |
| A829 | Rabies, unspecified | Inflammatory conditions of the brain |  |  | 0 |
| A83 | Mosquito-borne viral encephalitis | Inflammatory conditions of the brain |  |  | 0 |
| A830 | Japanese encephalitis | Inflammatory conditions of the brain |  |  | 0 |
| A831 | Western equine encephalitis | Inflammatory conditions of the brain |  |  | 0 |
| A832 | Eastern equine encephalitis | Inflammatory conditions of the brain |  |  | 0 |
| A833 | St Louis encephalitis | Inflammatory conditions of the brain |  |  | 0 |
| A834 | Australian encephalitis | Inflammatory conditions of the brain |  |  | 0 |
| A835 | California encephalitis | Inflammatory conditions of the brain |  |  | 0 |
| A836 | Rocio virus disease | Inflammatory conditions of the brain |  |  | 0 |
| A838 | Other mosquito-borne viral encephalitis | Inflammatory conditions of the brain |  |  | 0 |
| A839 | Mosquito-borne viral encephalitis, unspecified | Inflammatory conditions of the brain |  |  | 0 |
| A84 | Tick-borne viral encephalitis | Inflammatory conditions of the brain |  |  | 0 |
| A840 | Far Eastern tick-borne encephalitis [Russian spring-summer encephalitis] | Inflammatory conditions of the brain |  |  | 0 |
| A841 | Central European tick-borne encephalitis | Inflammatory conditions of the brain |  |  | 0 |
| A848 | Other tick-borne viral encephalitis | Inflammatory conditions of the brain |  |  | 0 |
| A849 | Tick-borne viral encephalitis, unspecified | Inflammatory conditions of the brain |  |  | 0 |
| A85 | Other viral encephalitis, not elsewhere classified | Inflammatory conditions of the brain |  |  | 0 |
| A850 | Enteroviral encephalitis | Inflammatory conditions of the brain |  |  | 0 |
| A851 | Adenoviral encephalitis | Inflammatory conditions of the brain |  |  | 0 |
| A852 | Arthropod-borne viral encephalitis, unspecified | Inflammatory conditions of the brain |  |  | 0 |
| A858 | Other specified viral encephalitis | Inflammatory conditions of the brain |  |  | 0 |
| A86 | Unspecified viral encephalitis | Inflammatory conditions of the brain |  |  | 0 |
| B003 | Herpesviral meningitis | Inflammatory conditions of the brain | Meningitis |  | 0 |
| B004 | Herpesviral encephalitis | Inflammatory conditions of the brain |  |  | 0 |
| B010 | Varicella meningitis | Inflammatory conditions of the brain | Meningitis |  | 0 |
| B011 | Varicella encephalitis | Inflammatory conditions of the brain |  |  | 0 |
| B020 | Zoster encephalitis | Inflammatory conditions of the brain |  |  | 0 |
| B021 | Zoster meningitis | Inflammatory conditions of the brain | Meningitis |  | 0 |
| B050 | Measles complicated by encephalitis | Inflammatory conditions of the brain |  |  | 0 |
| B051 | Measles complicated by meningitis | Inflammatory conditions of the brain | Meningitis |  | 0 |
| B060 | Rubella with neurological complications | Inflammatory conditions of the brain |  |  | 0 |
| B261 | Mumps meningitis | Inflammatory conditions of the brain | Meningitis |  | 0 |
| B262 | Mumps encephalitis | Inflammatory conditions of the brain |  |  | 0 |
| B375 | Candidal meningitis | Inflammatory conditions of the brain | Meningitis |  | 0 |
| B384 | Coccidioidomycosis meningitis | Inflammatory conditions of the brain | Meningitis |  | 0 |
| B431 | Phaeomycotic brain abscess | Inflammatory conditions of the brain |  |  | 0 |
| B451 | Cerebral cryptococcosis | Inflammatory conditions of the brain |  |  | 0 |
| B500 | Plasmodium falciparum malaria with cerebral complications | Inflammatory conditions of the brain |  |  | 0 |
| B582 | Toxoplasma meningoencephalitis | Inflammatory conditions of the brain |  |  | 0 |
| B690 | Cysticercosis of central nervous system | Inflammatory conditions of the brain |  |  | 0 |
| B900 | Sequelae of central nervous system tuberculosis | Inflammatory conditions of the brain |  |  | 0 |
| B91 | Sequelae of poliomyelitis | Inflammatory conditions of the brain |  |  | 0 |
| B941 | Sequelae of viral encephalitis | Inflammatory conditions of the brain |  |  | 0 |
| G00 | Bacterial meningitis, not elsewhere classified | Inflammatory conditions of the brain | Meningitis |  | 0 |
| G000 | Haemophilus meningitis | Inflammatory conditions of the brain | Meningitis |  | 0 |
| G001 | Pneumococcal meningitis | Inflammatory conditions of the brain | Meningitis |  | 0 |
| G002 | Streptococcal meningitis | Inflammatory conditions of the brain | Meningitis |  | 0 |
| G003 | Staphylococcal meningitis | Inflammatory conditions of the brain | Meningitis |  | 0 |
| G008 | Other bacterial meningitis | Inflammatory conditions of the brain | Meningitis |  | 0 |
| G009 | Bacterial meningitis, unspecified | Inflammatory conditions of the brain | Meningitis |  | 0 |
| G01 | Meningitis in bacterial diseases classified elsewhere | Inflammatory conditions of the brain | Meningitis |  | 0 |
| G021 | Meningitis in mycoses | Inflammatory conditions of the brain | Meningitis |  | 0 |
| G028 | Meningitis in other specified infectious and parasitic diseases classified elsewhere | Inflammatory conditions of the brain | Meningitis |  | 0 |
| G03 | Meningitis due to other and unspecified causes | Inflammatory conditions of the brain | Meningitis |  | 0 |
| G030 | Nonpyogenic meningitis | Inflammatory conditions of the brain | Meningitis |  | 0 |
| G031 | Chronic meningitis | Inflammatory conditions of the brain | Meningitis |  | 0 |
| G032 | Benign recurrent meningitis [Mollaret] | Inflammatory conditions of the brain | Meningitis |  | 0 |
| G038 | Meningitis due to other specified causes | Inflammatory conditions of the brain | Meningitis |  | 0 |
| G039 | Meningitis, unspecified | Inflammatory conditions of the brain | Meningitis |  | 0 |
| G04 | Encephalitis, myelitis and encephalomyelitis | Inflammatory conditions of the brain |  |  | 0 |
| G040 | Acute disseminated encephalitis | Inflammatory conditions of the brain |  |  | 0 |
| G041 | Tropical spastic paraplegia | Inflammatory conditions of the brain |  |  | 0 |
| G042 | Bacterial meningoencephalitis and meningomyelitis, not elsewhere classified | Inflammatory conditions of the brain |  |  | 0 |
| G048 | Other encephalitis, myelitis and encephalomyelitis | Inflammatory conditions of the brain |  |  | 0 |
| G049 | Encephalitis, myelitis and encephalomyelitis, unspecified | Inflammatory conditions of the brain |  |  | 0 |
| G05 | Encephalitis, myelitis and encephalomyelitis in diseases classified elsewhere | Inflammatory conditions of the brain |  |  | 0 |
| G050 | Encephalitis, myelitis and encephalomyelitis in bacterial diseases classified elsewhere | Inflammatory conditions of the brain |  |  | 0 |
| G051 | Encephalitis, myelitis and encephalomyelitis in viral diseases classified elsewhere | Inflammatory conditions of the brain |  |  | 0 |
| G052 | Encephalitis, myelitis and encephalomyelitis in other infectious and parasitic diseases classified elsewhere | Inflammatory conditions of the brain |  |  | 0 |
| G058 | Encephalitis, myelitis and encephalomyelitis in other diseases classified elsewhere | Inflammatory conditions of the brain |  |  | 0 |
| G06 | Intracranial and intraspinal abscess and granuloma | Inflammatory conditions of the brain |  |  | 0 |
| G060 | Intracranial abscess and granuloma | Inflammatory conditions of the brain |  |  | 0 |
| G061 | Intraspinal abscess and granuloma | Inflammatory conditions of the brain |  |  | 0 |
| G062 | Extradural and subdural abscess, unspecified | Inflammatory conditions of the brain |  |  | 0 |
| G07 | Intracranial and intraspinal abscess and granuloma in diseases classified elsewhere | Inflammatory conditions of the brain |  |  | 0 |
| G08 | Intracranial and intraspinal phlebitis and thrombophlebitis | Inflammatory conditions of the brain |  |  | 0 |
| G09 | Sequelae of inflammatory diseases of central nervous system | Inflammatory conditions of the brain |  |  | 0 |
| M896 | Osteopathy after poliomyelitis | Inflammatory conditions of the brain |  |  | 0 |
| G92 | Toxic encephalopathy |  |  |  | 0 |
| G930 | Cerebral cysts |  |  |  | 0 |
| G931 | Anoxic brain damage, not elsewhere classified |  |  |  | 0 |
| G934 | Encephalopathy, unspecified |  |  |  | 0 |
| G935 | Compression of brain |  |  |  | 0 |
| G936 | Cerebral oedema |  |  |  | 0 |
| G936 | Cerebral oedema |  |  |  | 0 |
| G937 | Reye syndrome |  |  |  | 0 |
| G938 | Other specified disorders of brain |  |  |  | 0 |
| G939 | Disorder of brain, unspecified |  |  |  | 0 |
| G94 | Other disorders of brain in diseases classified elsewhere |  |  |  | 0 |
| G948 | Other specified disorders of brain in diseases classified elsewhere |  |  |  | 0 |
|  | | | | | |
| **Sensory impairments** | | | | | |
| H903 | Sensorineural hearing loss, bilateral | Hearing impairment |  |  | 0 |
| H906 | Mixed conductive and sensorineural hearing loss, bilateral | Hearing impairment |  |  | 0 |
| H913 | Deaf mutism, not elsewhere classified | Hearing impairment |  |  | 0 |
| Z453 | Adjustment and management of implanted hearing device | Hearing impairment |  |  | 0 |
| Z461 | Fitting and adjustment of hearing aid | Hearing impairment |  |  | 0 |
| Z962 | Presence of otological and audiological implants | Hearing impairment |  |  | 0 |
| Z974 | Presence of external hearing-aid | Hearing impairment |  |  | 0 |
| H904 | Sensorineural hearing loss, unilateral with unrestricted hearing on the contralateral side | Hearing impairment |  |  | 0 |
| H905 | Sensorineural hearing loss, unspecified | Hearing impairment |  |  | 0 |
| H907 | Mixed conductive and sensorineural hearing loss, unilateral with unrestricted hearing on the contralateral side | Hearing impairment |  |  | 0 |
| H908 | Mixed conductive and sensorineural hearing loss, unspecified | Hearing impairment |  |  | 0 |
| H540 | Blindness, binocular | Visual impairment |  |  | 0 |
| H541 | Severe visual impairment, binocular | Visual impairment |  |  | 0 |
| H542 | Moderate visual impairment, binocular | Visual impairment |  |  | 0 |
| H351 | Retinopathy of prematurity | Visual impairment |  |  | 0 |
| H185 | Hereditary corneal dystrophies | Visual impairment |  |  | 0 |
| H312 | Hereditary choroidal dystrophy | Visual impairment |  |  | 0 |
| H360 | Diabetic retinopathy | Visual impairment |  |  | 0 |
| H368 | Other retinal disorders in diseases classified elsewhere | Visual impairment |  |  | 0 |
| H472 | Optic atrophy | Visual impairment |  |  | 0 |
| H473 | Other disorders of optic disc | Visual impairment |  |  | 0 |
| H474 | Disorders of optic chiasm | Visual impairment |  |  | 0 |
| H475 | Disorders of other visual pathways | Visual impairment |  |  | 0 |
| H476 | Disorders of visual cortex | Visual impairment |  |  | 0 |
| H477 | Disorder of visual pathways, unspecified | Visual impairment |  |  | 0 |
| H480 | Optic atrophy in diseases classified elsewhere | Visual impairment |  |  | 0 |
| H488 | Other disorders of optic nerve and visual pathways in diseases classified elsewhere | Visual impairment |  |  | 0 |
| H545 | Severe visual impairment, monocular | Visual impairment |  |  | 0 |
| H549 | Unspecified visual impairment (binocular) | Visual impairment |  |  | 0 |
| Q134 | Other congenital corneal malformations | Visual impairment |  |  | 0 |
| Q138 | Other congenital malformations of anterior segment of eye | Visual impairment |  |  | 0 |
| Q139 | Congenital malformation of anterior segment of eye, unspecified | Visual impairment |  |  | 0 |
| Q150 | Congenital glaucoma | Visual impairment |  |  | 0 |
|  | | | | | |
| **Impairment of motor function** | | | | | |
| A810 | Creutzfeldt-Jakob disease |  |  |  | 0 |
| A811 | Subacute sclerosing panencephalitis |  |  |  | 0 |
| A812 | Progressive multifocal leukoencephalopathy |  |  |  | 0 |
| A818 | Other atypical virus infections of central nervous system |  |  |  | 0 |
| G10 | Huntington disease |  |  |  | 0 |
| G11 | Hereditary ataxia |  |  |  | 0 |
| G110 | Congenital nonprogressive ataxia |  |  |  | 0 |
| G111 | Early-onset cerebellar ataxia |  |  |  | 0 |
| G112 | Late-onset cerebellar ataxia |  |  |  | 0 |
| G113 | Cerebellar ataxia with defective DNA repair |  |  |  | 0 |
| G114 | Hereditary spastic paraplegia |  |  |  | 0 |
| G118 | Other hereditary ataxias |  |  |  | 0 |
| G119 | Hereditary ataxia, unspecified |  |  |  | 0 |
| G12 | Spinal muscular atrophy and related syndromes |  |  |  | 0 |
| G120 | Infantile spinal muscular atrophy, type I [Werdnig-Hoffman] |  |  |  | 0 |
| G121 | Other inherited spinal muscular atrophy |  |  |  | 0 |
| G122 | Motor neuron disease |  |  |  | 0 |
| G128 | Other spinal muscular atrophies and related syndromes |  |  |  | 0 |
| G129 | Spinal muscular atrophy, unspecified |  |  |  | 0 |
| G13 | Systemic atrophies primarily affecting central nervous system in diseases classified elsewhere |  |  |  | 0 |
| G130 | Paraneoplastic neuromyopathy and neuropathy |  |  |  | 0 |
| G130 | Paraneoplastic neuromyopathy and neuropathy |  |  |  | 0 |
| G131 | Other systemic atrophy primarily affecting central nervous system in neoplastic disease |  |  |  | 0 |
| G132 | Systemic atrophy primarily affecting central nervous system in myxoedema |  |  |  | 0 |
| G138 | Systemic atrophy primarily affecting central nervous system in other diseases classified elsewhere |  |  |  | 0 |
| G14 | Postpolio syndrome |  |  |  | 0 |
| G35 | Multiple sclerosis |  |  |  | 0 |
| G36 | Other acute disseminated demyelination |  |  |  | 0 |
| G360 | Neuromyelitis optica [Devic] |  |  |  | 0 |
| G361 | Acute and subacute haemorrhagic leukoencephalitis [Hurst] |  |  |  | 0 |
| G368 | Other specified acute disseminated demyelination |  |  |  | 0 |
| G369 | Acute disseminated demyelination, unspecified |  |  |  | 0 |
| G37 | Other demyelinating diseases of central nervous system |  |  |  | 0 |
| G370 | Diffuse sclerosis |  |  |  | 0 |
| G371 | Central demyelination of corpus callosum |  |  |  | 0 |
| G372 | Central pontine myelinolysis |  |  |  | 0 |
| G373 | Acute transverse myelitis in demyelinating disease of central nervous system |  |  |  | 0 |
| G374 | Subacute necrotizing myelitis |  |  |  | 0 |
| G375 | Concentric sclerosis [BalÃ³] |  |  |  | 0 |
| G378 | Other specified demyelinating diseases of central nervous system |  |  |  | 0 |
| G379 | Demyelinating disease of central nervous system, unspecified |  |  |  | 0 |
| G70 | Myasthenia gravis and other myoneural disorders |  |  |  | 0 |
| G700 | Myasthenia gravis |  |  |  | 0 |
| G701 | Toxic myoneural disorders |  |  |  | 0 |
| G702 | Congenital and developmental myasthenia |  |  |  | 0 |
| G708 | Other specified myoneural disorders |  |  |  | 0 |
| G709 | Myoneural disorder, unspecified |  |  |  | 0 |
| G71 | Primary disorders of muscles |  |  |  | 0 |
| G710 | Muscular dystrophy |  |  |  | 0 |
| G711 | Myotonic disorders |  |  |  | 0 |
| G712 | Congenital myopathies |  |  |  | 0 |
| G713 | Mitochondrial myopathy, not elsewhere classified |  |  |  | 0 |
| G718 | Other primary disorders of muscles |  |  |  | 0 |
| G719 | Primary disorder of muscle, unspecified |  |  |  | 0 |
| G723 | Periodic paralysis |  |  |  | 0 |
| G724 | Inflammatory myopathy, not elsewhere classified |  |  |  | 0 |
| G728 | Other specified myopathies |  |  |  | 0 |
| G729 | Myopathy, unspecified |  |  |  | 0 |
| G73 | Disorders of myoneural junction and muscle in diseases classified elsewhere |  |  |  | 0 |
| G730 | Myasthenic syndromes in endocrine diseases |  |  |  | 0 |
| G731 | Lambert-Eaton syndrome |  |  |  | 0 |
| G732 | Other myasthenic syndromes in neoplastic disease |  |  |  | 0 |
| G733 | Myasthenic syndromes in other diseases classified elsewhere |  |  |  | 0 |
| G734 | Myopathy in infectious and parasitic diseases classified elsewhere |  |  |  | 0 |
| G735 | Myopathy in endocrine diseases |  |  |  | 0 |
| G736 | Myopathy in metabolic diseases |  |  |  | 0 |
| G737 | Myopathy in other diseases classified elsewhere |  |  |  | 0 |
| G20 | Parkinson disease |  |  |  | 0 |
| G21 | Secondary parkinsonism |  |  |  | 0 |
| G210 | Malignant neuroleptic syndrome |  |  |  | 0 |
| G211 | Other drug-induced secondary parkinsonism |  |  |  | 0 |
| G212 | Secondary parkinsonism due to other external agents |  |  |  | 0 |
| G213 | Postencephalitic parkinsonism |  |  |  | 0 |
| G214 | Vascular parkinsonism |  |  |  | 0 |
| G218 | Other secondary parkinsonism |  |  |  | 0 |
| G219 | Secondary parkinsonism, unspecified |  |  |  | 0 |
| G22 | Parkinsonism in diseases classified elsewhere |  |  |  | 0 |
| G23 | Other degenerative diseases of basal ganglia |  |  |  | 0 |
| G230 | Hallervorden-Spatz disease |  |  |  | 0 |
| G231 | Progressive supranuclear ophthalmoplegia [Steele-Richardson-Olszewski] |  |  |  | 0 |
| G232 | Multiple system atrophy, parkinsonian type [MSA-P] |  |  |  | 0 |
| G233 | Multiple system atrophy, cerebellar type [MSA-C] |  |  |  | 0 |
| G238 | Other specified degenerative diseases of basal ganglia |  |  |  | 0 |
| G239 | Degenerative disease of basal ganglia, unspecified |  |  |  | 0 |
| G240 | Drug-induced dystonia |  |  |  | 0 |
| G241 | Idiopathic familial dystonia |  |  |  | 0 |
| G242 | Idiopathic nonfamilial dystonia |  |  |  | 0 |
| G243 | Spasmodic torticollis |  |  |  | 0 |
| G244 | Idiopathic orofacial dystonia |  |  |  | 0 |
| G248 | Other dystonia |  |  |  | 0 |
| G249 | Dystonia, unspecified |  |  |  | 0 |
| G252 | Other specified forms of tremor |  |  |  | 0 |
| G254 | Drug-induced chorea |  |  |  | 0 |
| G255 | Other chorea |  |  |  | 0 |
| G258 | Other specified extrapyramidal and movement disorders |  |  |  | 0 |
| G259 | Extrapyramidal and movement disorder, unspecified |  |  |  | 0 |
| G26 | Extrapyramidal and movement disorders in diseases classified elsewhere |  |  |  | 0 |
| Y467 | Antiparkinsonism drugs |  |  |  | 0 |
| Y468 | Antispasticity drugs |  |  |  | 0 |
| G60 | Hereditary and idiopathic neuropathy |  |  |  | 0 |
| G600 | Hereditary motor and sensory neuropathy |  |  |  | 0 |
| G601 | Refsum disease |  |  |  | 0 |
| G602 | Neuropathy in association with hereditary ataxia |  |  |  | 0 |
| G603 | Idiopathic progressive neuropathy |  |  |  | 0 |
| G608 | Other hereditary and idiopathic neuropathies |  |  |  | 0 |
| G609 | Hereditary and idiopathic neuropathy, unspecified |  |  |  | 0 |
| G611 | Serum neuropathy |  |  |  | 0 |
| G618 | Other inflammatory polyneuropathies |  |  |  | 0 |
| G619 | Inflammatory polyneuropathy, unspecified |  |  |  | 0 |
| G62 | Other polyneuropathies |  |  |  | 0 |
| G620 | Drug-induced polyneuropathy |  |  |  | 0 |
| G621 | Alcoholic polyneuropathy |  |  |  | 0 |
| G622 | Polyneuropathy due to other toxic agents |  |  |  | 0 |
| G628 | Other specified polyneuropathies |  |  |  | 0 |
| G629 | Polyneuropathy, unspecified |  |  |  | 0 |
| G63 | Polyneuropathy in diseases classified elsewhere |  |  |  | 0 |
| G630 | Polyneuropathy in infectious and parasitic diseases classified elsewhere |  |  |  | 0 |
| G631 | Polyneuropathy in neoplastic disease |  |  |  | 0 |
| G632 | Diabetic polyneuropathy |  |  |  | 0 |
| G633 | Polyneuropathy in other endocrine and metabolic diseases |  |  |  | 0 |
| G634 | Polyneuropathy in nutritional deficiency |  |  |  | 0 |
| G635 | Polyneuropathy in systemic connective tissue disorders |  |  |  | 0 |
| G636 | Polyneuropathy in other musculoskeletal disorders |  |  |  | 0 |
| G638 | Polyneuropathy in other diseases classified elsewhere |  |  |  | 0 |
| G31 | Other degenerative diseases of nervous system, not elsewhere classified |  |  |  | 0 |
| G310 | Circumscribed brain atrophy |  |  |  | 0 |
| G311 | Senile degeneration of brain, not elsewhere classified |  |  |  | 0 |
| G312 | Degeneration of nervous system due to alcohol |  |  |  | 0 |
| G318 | Other specified degenerative diseases of nervous system |  |  |  | 0 |
| G319 | Degenerative disease of nervous system, unspecified |  |  |  | 0 |
| G32 | Other degenerative disorders of nervous system in diseases classified elsewhere |  |  |  | 0 |
| G320 | Subacute combined degeneration of spinal cord in diseases classified elsewhere |  |  |  | 0 |
| G328 | Other specified degenerative disorders of nervous system in diseases classified elsewhere |  |  |  | 0 |
| G834 | Cauda equina syndrome |  |  |  | 0 |
| G835 | Locked-in syndrome |  |  |  | 0 |
| G838 | Other specified paralytic syndromes |  |  |  | 0 |
| G839 | Paralytic syndrome, unspecified |  |  |  | 0 |
| G900 | Idiopathic peripheral autonomic neuropathy |  |  |  | 0 |
| G901 | Familial dysautonomia [Riley-Day] |  |  |  | 0 |
| G904 | Autonomic dysreflexia |  |  |  | 0 |
| G908 | Other disorders of autonomic nervous system |  |  |  | 0 |
| G909 | Disorder of autonomic nervous system, unspecified |  |  |  | 0 |
| G95 | Other diseases of spinal cord |  |  |  | 0 |
| G950 | Syringomyelia and syringobulbia |  |  |  | 0 |
| G951 | Vascular myelopathies |  |  |  | 0 |
| G951 | Vascular myelopathies |  |  |  | 0 |
| G952 | Cord compression, unspecified |  |  |  | 0 |
| G958 | Other specified diseases of spinal cord |  |  |  | 0 |
| G959 | Disease of spinal cord, unspecified |  |  |  | 0 |
| G99 | Other disorders of nervous system in diseases classified elsewhere |  |  |  | 0 |
| G990 | Autonomic neuropathy in endocrine and metabolic diseases |  |  |  | 0 |
| G991 | Other disorders of autonomic nervous system in other diseases classified elsewhere |  |  |  | 0 |
| G992 | Myelopathy in diseases classified elsewhere |  |  |  | 0 |
| G998 | Other specified disorders of nervous system in diseases classified elsewhere |  |  |  | 0 |
|  | | | | | |
| **Perinatal conditions** | | | | | |
| P210 | Severe birth asphyxia | Severe birth asphyxia |  |  | 0 |
| P57 | Kernicterus | Perinatal Brain Damage |  |  | 0 |
| P570 | Kernicterus due to isoimmunization | Perinatal Brain Damage |  |  | 0 |
| P578 | Other specified kernicterus | Perinatal Brain Damage |  |  | 0 |
| P579 | Kernicterus, unspecified | Perinatal Brain Damage |  |  | 0 |
| P90 | Convulsions of newborn | Perinatal Brain Damage |  |  | 0 |
| P912 | Neonatal cerebral leukomalacia | Perinatal Brain Damage |  |  | 0 |
| G46 | Vascular syndromes of brain in cerebrovascular diseases | Perinatal Brain Damage | Perinatal stroke |  | 1 |
| G460 | Middle cerebral artery syndrome | Perinatal Brain Damage | Perinatal stroke |  | 1 |
| G461 | Anterior cerebral artery syndrome | Perinatal Brain Damage | Perinatal stroke |  | 1 |
| G462 | Posterior cerebral artery syndrome | Perinatal Brain Damage | Perinatal stroke |  | 1 |
| G463 | Brain stem stroke syndrome | Perinatal Brain Damage | Perinatal stroke |  | 1 |
| G464 | Cerebellar stroke syndrome | Perinatal Brain Damage | Perinatal stroke |  | 1 |
| G465 | Pure motor lacunar syndrome | Perinatal Brain Damage | Perinatal stroke |  | 1 |
| G466 | Pure sensory lacunar syndrome | Perinatal Brain Damage | Perinatal stroke |  | 1 |
| G467 | Other lacunar syndromes | Perinatal Brain Damage | Perinatal stroke |  | 1 |
| G468 | Other vascular syndromes of brain in cerebrovascular diseases | Perinatal Brain Damage | Perinatal stroke |  | 1 |
| I60 | Subarachnoid haemorrhage | Perinatal Brain Damage | Perinatal stroke |  | 1 |
| I600 | Subarachnoid haemorrhage from carotid siphon and bifurcation | Perinatal Brain Damage | Perinatal stroke |  | 1 |
| I601 | Subarachnoid haemorrhage from middle cerebral artery | Perinatal Brain Damage | Perinatal stroke |  | 1 |
| I602 | Subarachnoid haemorrhage from anterior communicating artery | Perinatal Brain Damage | Perinatal stroke |  | 1 |
| I603 | Subarachnoid haemorrhage from posterior communicating artery | Perinatal Brain Damage | Perinatal stroke |  | 1 |
| I604 | Subarachnoid haemorrhage from basilar artery | Perinatal Brain Damage | Perinatal stroke |  | 1 |
| I605 | Subarachnoid haemorrhage from vertebral artery | Perinatal Brain Damage | Perinatal stroke |  | 1 |
| I606 | Subarachnoid haemorrhage from other intracranial arteries | Perinatal Brain Damage | Perinatal stroke |  | 1 |
| I607 | Subarachnoid haemorrhage from intracranial artery, unspecified | Perinatal Brain Damage | Perinatal stroke |  | 1 |
| I608 | Other subarachnoid haemorrhage | Perinatal Brain Damage | Perinatal stroke |  | 1 |
| I609 | Subarachnoid haemorrhage, unspecified | Perinatal Brain Damage | Perinatal stroke |  | 1 |
| I61 | Intracerebral haemorrhage | Perinatal Brain Damage | Perinatal stroke |  | 1 |
| I610 | Intracerebral haemorrhage in hemisphere, subcortical | Perinatal Brain Damage | Perinatal stroke |  | 1 |
| I611 | Intracerebral haemorrhage in hemisphere, cortical | Perinatal Brain Damage | Perinatal stroke |  | 1 |
| I612 | Intracerebral haemorrhage in hemisphere, unspecified | Perinatal Brain Damage | Perinatal stroke |  | 1 |
| I613 | Intracerebral haemorrhage in brain stem | Perinatal Brain Damage | Perinatal stroke |  | 1 |
| I614 | Intracerebral haemorrhage in cerebellum | Perinatal Brain Damage | Perinatal stroke |  | 1 |
| I615 | Intracerebral haemorrhage, intraventricular | Perinatal Brain Damage | Perinatal stroke |  | 1 |
| I616 | Intracerebral haemorrhage, multiple localized | Perinatal Brain Damage | Perinatal stroke |  | 1 |
| I618 | Other intracerebral haemorrhage | Perinatal Brain Damage | Perinatal stroke |  | 1 |
| I619 | Intracerebral haemorrhage, unspecified | Perinatal Brain Damage | Perinatal stroke |  | 1 |
| I63 | Cerebral infarction | Perinatal Brain Damage | Perinatal stroke |  | 1 |
| I630 | Cerebral infarction due to thrombosis of precerebral arteries | Perinatal Brain Damage | Perinatal stroke |  | 1 |
| I631 | Cerebral infarction due to embolism of precerebral arteries | Perinatal Brain Damage | Perinatal stroke |  | 1 |
| I632 | Cerebral infarction due to unspecified occlusion or stenosis of precerebral arteries | Perinatal Brain Damage | Perinatal stroke |  | 1 |
| I633 | Cerebral infarction due to thrombosis of cerebral arteries | Perinatal Brain Damage | Perinatal stroke |  | 1 |
| I634 | Cerebral infarction due to embolism of cerebral arteries | Perinatal Brain Damage | Perinatal stroke |  | 1 |
| I635 | Cerebral infarction due to unspecified occlusion or stenosis of cerebral arteries | Perinatal Brain Damage | Perinatal stroke |  | 1 |
| I636 | Cerebral infarction due to cerebral venous thrombosis, nonpyogenic | Perinatal Brain Damage | Perinatal stroke |  | 1 |
| I638 | Other cerebral infarction | Perinatal Brain Damage | Perinatal stroke |  | 1 |
| I639 | Cerebral infarction, unspecified | Perinatal Brain Damage | Perinatal stroke |  | 1 |
| I64 | Stroke, not specified as haemorrhage or infarction | Perinatal Brain Damage | Perinatal stroke |  | 1 |
| I670 | Dissection of cerebral arteries, nonruptured | Perinatal Brain Damage | Perinatal stroke |  | 1 |
| I671 | Cerebral aneurysm, nonruptured | Perinatal Brain Damage | Perinatal stroke |  | 1 |
| I673 | Progressive vascular leukoencephalopathy | Perinatal Brain Damage | Perinatal stroke |  | 1 |
| I675 | Moyamoya disease | Perinatal Brain Damage | Perinatal stroke |  | 1 |
| I676 | Nonpyogenic thrombosis of intracranial venous system | Perinatal Brain Damage | Perinatal stroke |  | 1 |
| I677 | Cerebral arteritis, not elsewhere classified | Perinatal Brain Damage | Perinatal stroke |  | 1 |
| I678 | Other specified cerebrovascular diseases | Perinatal Brain Damage | Perinatal stroke |  | 1 |
| I679 | Cerebrovascular disease, unspecified | Perinatal Brain Damage | Perinatal stroke |  | 1 |
| I690 | Sequelae of subarachnoid haemorrhage | Perinatal Brain Damage | Perinatal stroke |  | 1 |
| I691 | Sequelae of intracerebral haemorrhage | Perinatal Brain Damage | Perinatal stroke |  | 1 |
| I693 | Sequelae of cerebral infarction | Perinatal Brain Damage | Perinatal stroke |  | 1 |
| I694 | Sequelae of stroke, not specified as haemorrhage or infarction | Perinatal Brain Damage | Perinatal stroke |  | 1 |
| P524 | Intracerebral (nontraumatic) haemorrhage of fetus and newborn | Perinatal Brain Damage | Perinatal stroke |  | 0 |
| P525 | Subarachnoid (nontraumatic) haemorrhage of fetus and newborn | Perinatal Brain Damage | Perinatal stroke |  | 0 |
| P526 | Cerebellar (nontraumatic) and posterior fossa haemorrhage of fetus and newborn | Perinatal Brain Damage | Perinatal stroke |  | 0 |
| P910 | Neonatal cerebral ischaemia | Perinatal Brain Damage | Perinatal stroke |  | 0 |
| P911 | Acquired periventricular cysts of newborn | Perinatal Brain Damage | Perinatal stroke |  | 0 |
| G91 | Hydrocephalus | Perinatal Brain Damage | Intracranial haemorrhage |  | 1 |
| G910 | Communicating hydrocephalus | Perinatal Brain Damage | Intracranial haemorrhage |  | 1 |
| G911 | Obstructive hydrocephalus | Perinatal Brain Damage | Intracranial haemorrhage |  | 1 |
| G912 | Normal-pressure hydrocephalus | Perinatal Brain Damage | Intracranial haemorrhage |  | 1 |
| G913 | Post-traumatic hydrocephalus, unspecified | Perinatal Brain Damage | Intracranial haemorrhage |  | 1 |
| G918 | Other hydrocephalus | Perinatal Brain Damage | Intracranial haemorrhage |  | 1 |
| G919 | Hydrocephalus, unspecified | Perinatal Brain Damage | Intracranial haemorrhage |  | 1 |
| G940 | Hydrocephalus in infectious and parasitic diseases classified elsewhere | Perinatal Brain Damage | Intracranial haemorrhage |  | 1 |
| G941 | Hydrocephalus in neoplastic disease | Perinatal Brain Damage | Intracranial haemorrhage |  | 1 |
| G942 | Hydrocephalus in other diseases classified elsewhere | Perinatal Brain Damage | Intracranial haemorrhage |  | 1 |
| G972 | Intracranial hypotension following ventricular shunting | Perinatal Brain Damage | Intracranial haemorrhage |  | 1 |
| P100 | Subdural haemorrhage due to birth injury | Perinatal Brain Damage | Intracranial haemorrhage |  | 0 |
| P101 | Cerebral haemorrhage due to birth injury | Perinatal Brain Damage | Intracranial haemorrhage |  | 0 |
| P102 | Intraventricular haemorrhage due to birth injury | Perinatal Brain Damage | Intracranial haemorrhage |  | 0 |
| P103 | Subarachnoid haemorrhage due to birth injury | Perinatal Brain Damage | Intracranial haemorrhage |  | 0 |
| P104 | Tentorial tear due to birth injury | Perinatal Brain Damage | Intracranial haemorrhage |  | 0 |
| P108 | Other intracranial lacerations and haemorrhages due to birth injury | Perinatal Brain Damage | Intracranial haemorrhage |  | 0 |
| P109 | Unspecified intracranial laceration and haemorrhage due to birth injury | Perinatal Brain Damage | Intracranial haemorrhage |  | 0 |
| P522 | Intraventricular (nontraumatic) haemorrhage, grade 3 and grade 4, of fetus and newborn | Perinatal Brain Damage | Intracranial haemorrhage |  | 0 |
| P523 | Unspecified intraventricular (nontraumatic) haemorrhage of fetus and newborn | Perinatal Brain Damage | Intracranial haemorrhage |  | 0 |
| P528 | Other intracranial (nontraumatic) haemorrhages of fetus and newborn | Perinatal Brain Damage | Intracranial haemorrhage |  | 0 |
| P529 | Intracranial (nontraumatic) haemorrhage of fetus and newborn, unspecified | Perinatal Brain Damage | Intracranial haemorrhage |  | 0 |
| P917 | Acquired hydrocephalus of newborn | Perinatal Brain Damage | Intracranial haemorrhage |  | 0 |
| T850 | Mechanical complication of ventricular intracranial (communicating) shunt | Perinatal Brain Damage | Intracranial haemorrhage |  | 1 |
| Z982 | Presence of cerebrospinal fluid drainage device | Perinatal Brain Damage | Intracranial haemorrhage |  | 1 |
| P915 | Neonatal coma | Perinatal Brain Damage |  |  | 0 |
| P916 | Hypoxic ischaemic encephalopathy of newborn | Perinatal Brain Damage |  |  | 0 |
| A170 | Tuberculous meningitis | Perinatal Brain Damage | CNS infection |  | 1 |
| A171 | Meningeal tuberculoma | Perinatal Brain Damage | CNS infection |  | 1 |
| A203 | Plague meningitis | Perinatal Brain Damage | CNS infection |  | 1 |
| A321 | Listerial meningitis and meningoencephalitis | Perinatal Brain Damage | CNS infection |  | 1 |
| A390 | Meningococcal meningitis | Perinatal Brain Damage | CNS infection |  | 1 |
| A872 | Lymphocytic choriomeningitis | Perinatal Brain Damage | CNS infection |  | 1 |
| B003 | Herpesviral meningitis | Perinatal Brain Damage | CNS infection |  | 1 |
| B004 | Herpesviral encephalitis | Perinatal Brain Damage | CNS infection |  | 1 |
| B007 | Disseminated herpesviral disease | Perinatal Brain Damage | CNS infection |  | 1 |
| B010 | Varicella meningitis | Perinatal Brain Damage | CNS infection |  | 1 |
| B011 | Varicella encephalitis | Perinatal Brain Damage | CNS infection |  | 1 |
| B018 | Varicella with other complications | Perinatal Brain Damage | CNS infection |  | 1 |
| B375 | Candidal meningitis | Perinatal Brain Damage | CNS infection |  | 1 |
| G00 | Bacterial meningitis, not elsewhere classified | Perinatal Brain Damage | CNS infection |  | 1 |
| G000 | Haemophilus meningitis | Perinatal Brain Damage | CNS infection |  | 1 |
| G001 | Pneumococcal meningitis | Perinatal Brain Damage | CNS infection |  | 1 |
| G002 | Streptococcal meningitis | Perinatal Brain Damage | CNS infection |  | 1 |
| G003 | Staphylococcal meningitis | Perinatal Brain Damage | CNS infection |  | 1 |
| G008 | Other bacterial meningitis | Perinatal Brain Damage | CNS infection |  | 1 |
| G009 | Bacterial meningitis, unspecified | Perinatal Brain Damage | CNS infection |  | 1 |
| G01 | Meningitis in bacterial diseases classified elsewhere | Perinatal Brain Damage | CNS infection |  | 1 |
| G021 | Meningitis in mycoses | Perinatal Brain Damage | CNS infection |  | 1 |
| G028 | Meningitis in other specified infectious and parasitic diseases classified elsewhere | Perinatal Brain Damage | CNS infection |  | 1 |
| G03 | Meningitis due to other and unspecified causes | Perinatal Brain Damage | CNS infection |  | 1 |
| G030 | Nonpyogenic meningitis | Perinatal Brain Damage | CNS infection |  | 1 |
| G031 | Chronic meningitis | Perinatal Brain Damage | CNS infection |  | 1 |
| G032 | Benign recurrent meningitis [Mollaret] | Perinatal Brain Damage | CNS infection |  | 1 |
| G038 | Meningitis due to other specified causes | Perinatal Brain Damage | CNS infection |  | 1 |
| G039 | Meningitis, unspecified | Perinatal Brain Damage | CNS infection |  | 1 |
| G05 | Encephalitis, myelitis and encephalomyelitis in diseases classified elsewhere | Perinatal Brain Damage | CNS infection |  | 1 |
| G050 | Encephalitis, myelitis and encephalomyelitis in bacterial diseases classified elsewhere | Perinatal Brain Damage | CNS infection |  | 1 |
| G051 | Encephalitis, myelitis and encephalomyelitis in viral diseases classified elsewhere | Perinatal Brain Damage | CNS infection |  | 1 |
| G052 | Encephalitis, myelitis and encephalomyelitis in other infectious and parasitic diseases classified elsewhere | Perinatal Brain Damage | CNS infection |  | 1 |
| G058 | Encephalitis, myelitis and encephalomyelitis in other diseases classified elsewhere | Perinatal Brain Damage | CNS infection |  | 1 |
| P352 | Congenital herpesviral [herpes simplex] infection | Perinatal Brain Damage | CNS infection |  | 0 |
| P044 | Fetus and newborn affected by maternal use of drugs of addiction |  |  |  | 0 |
| P961 | Neonatal withdrawal symptoms from maternal use of drugs of addiction |  |  |  | 0 |
| A50 | Congenital syphilis |  |  |  | 0 |
| A500 | Early congenital syphilis, symptomatic |  |  |  | 0 |
| A501 | Early congenital syphilis, latent |  |  |  | 0 |
| A502 | Early congenital syphilis, unspecified |  |  |  | 0 |
| A503 | Late congenital syphilitic oculopathy |  |  |  | 0 |
| A504 | Late congenital neurosyphilis [juvenile neurosyphilis] |  |  |  | 0 |
| A505 | Other late congenital syphilis, symptomatic |  |  |  | 0 |
| A506 | Late congenital syphilis, latent |  |  |  | 0 |
| A507 | Late congenital syphilis, unspecified |  |  |  | 0 |
| A509 | Congenital syphilis, unspecified |  |  |  | 0 |
| B060 | Rubella with neurological complications |  |  |  | 1 |
| B069 | Rubella without complication |  |  |  | 1 |
| B582 | Toxoplasma meningoencephalitis |  |  |  | 1 |
| P350 | Congenital rubella syndrome |  |  |  | 0 |
| P351 | Congenital cytomegalovirus infection |  |  |  | 0 |
| P358 | Other congenital viral diseases |  |  |  | 0 |
| P359 | Congenital viral disease, unspecified |  |  |  | 0 |
| P370 | Congenital tuberculosis |  |  |  | 0 |
| P371 | Congenital toxoplasmosis |  |  |  | 0 |

^†^ All codes contribute to classification of any neurodisability; ^‡^ Use of code is restricted to admissions occurring within the first 28 days of life.

ADHD: Attention Deficit Hyperactivity Disorder; CNS: Central Nervous System; ICD-10: International Classification of Diseases 10^th^ revision.

**Table S2. Special Educational Needs and Disability (SEND) types of need used to identify intellectual disability and autism in school records.**

| **Neurodisability** | **SEND type of need** | **Description** |
| --- | --- | --- |
| Intellectual disability  Note: Intellectual disability is commonly referred to as learning disability in England. | PMLD | **Profound & Multiple Learning Difficulty**  Where children are likely to have severe and complex learning difficulties as well as a physical disability or sensory impairment.^3^ |
|  | SLD | **Severe Learning Difficulty**  Where children are likely to need support in all areas of the curriculum and associated difficulties with mobility and communication.^3^ |
|  | Exclusions: MLD | **Moderate Learning Difficulty**  Excluded due to its status as a highly heterogeneous educational label that lacks clear diagnostic thresholds and frequently overlaps with general low academic attainment.^4^ |
| Autism | ASD | **Autistic Spectrum Disorder** Where children are likely to have particular difficulties with social interaction. They may also experience difficulties with language, communication and imagination, which can impact on how they relate to others.^3^ |

## References

1	Zylbersztejn A, Rees P, D’Souza R, Logan S, Cant A, Gimeno L, et al. Phenotyping Neurodisability in Hospital Records in England: A National Birth Cohort Using Linked Administrative Data. *Paediatric and Perinatal Epidemiology* 2025.

2	Zylbersztejn A, Stilwell PA, Zhu H, Ainsworth V, Allister J, Horridge K, et al. Trends in hospital admissions during transition from paediatric to adult services for young people with learning disabilities or autism: Population-based cohort study. *The Lancet Regional Health. Europe* 2023;24:100531.

3	Department for Education. *Special educational needs and disability code of practice: 0 to 25 years*.; 2015.

4	Norwich B, Ylonen ,Annamari, and Gwernan-Jones R. Moderate learning difficulties: searching for clarity and understanding. *Research Papers in Education* 2014;29:1–19.
